# HistoGWAS: an AI-enabled framework for automated genetic analysis of tissue phenotypes in histology cohorts

**DOI:** 10.1186/s13059-026-04031-z

**Published:** 2026-03-31

**Authors:** Shubham Chaudhary, Almut Voigts, Michael Bereket, Matthew L. Albert, Kristina Schwamborn, Eleftheria Zeggini, Francesco Paolo Casale

**Affiliations:** 1 Institute of AI for Health, Helmholtz Zentrum München – German Research Center for Environmental Health, Neuherberg, Germany; 2 Helmholtz Pioneer Campus, Helmholtz Zentrum München – German Research Center for Environmental Health, Neuherberg, Germany; 3 School of Computation, Information and Technology, Technical University of Munich, Garching, Germany; 4 TUM School of Medicine and Health, Technical University of Munich and Klinikum Rechts Der Isar, Munich, Germany; 5 Department of Computer Science, Stanford University, Stanford, CA USA; 6 Octant Biosciences, San Francisco, CA USA; 7 Institute of Pathology, TUM School of Medicine and Health, Technical University of Munich, Munich, Germany; 8 Institute of Translational Genomics, Helmholtz Zentrum München – German Research Center for Environmental Health, Neuherberg, Germany

**Keywords:** Histology, Genome-wide association studies, Variance component test, Semantic autoencoder, Kernel methods, Colocalization, Generative models

## Abstract

Understanding how genetic variation shapes tissue structure is crucial for disease biology, yet scalable, general-purpose frameworks for genetic analysis of histology traits are lacking. We present HistoGWAS, a framework for genome-wide association studies of histology data that leverages foundation models for automated trait definition, variance component models for efficient association testing, and generative models for variant effect interpretation. Applied to 11 tissues from the Genotype-Tissue Expression project, HistoGWAS identifies four genome-wide significant loci associated with tissue histology—tissue quantitative trait loci (tissueQTLs)—which we link to molecular changes and complex traits. Power analyses demonstrate scalability to population-scale histology cohorts.

**Supplementary Information:**

The online version contains supplementary material available at 10.1186/s13059-026-04031-z.

## Background

Genetic analysis of molecular, cellular, and tissue-level traits can clarify how disease-associated loci influence clinical outcomes by revealing the intermediate processes that mediate genetic effects. Initially focused on molecular traits such as gene expression and protein levels [1–7], these efforts have expanded to imaging-derived traits, advancing our understanding of disease biology and uncovering new biomarkers [8–15].

Histological images capture tissue architecture and cellular composition, revealing disease processes such as scarring and inflammation that may precede clinical manifestation [16, 17]. However, large-scale genetic studies of histological traits remain limited due to the high dimensionality and complexity of the data. Prior work has focused on targeted analyses [18, 19] or semi-quantitative scoring systems of disease severity, such as the NAFLD Activity Score in liver disease [20], whereas general frameworks for genome-wide genetic analysis of histology phenotypes are lacking.

Recent advances in artificial intelligence have transformed the analysis of complex biological data. Foundation models enable the derivation of quantitative embeddings from high-dimensional modalities, facilitating diverse downstream tasks [21–25]. Concurrently, generative models have improved our ability to model the effects of specific covariates on imaging and molecular traits [26–28]. However, despite substantial progress in AI-driven diagnostics and prognostics within computational pathology [29–34], frameworks that leverage AI for genome-wide genetic analysis of histology phenotypes remain lacking.

**Fig. 1:**
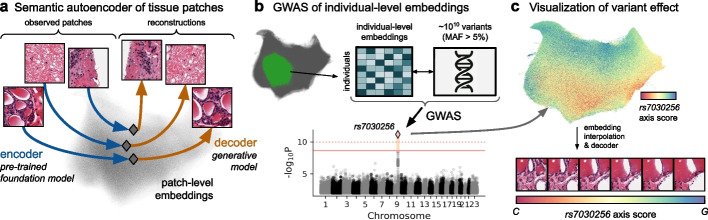
Overview of the HistoGWAS workflow. **a** A semantic autoencoder encodes slide patches into embeddings and reconstructs them into image space using a pretrained foundation model as the encoder and a generative model as the decoder. **b** Genome-wide association study (GWAS) of individual-level embeddings obtained by averaging patch embeddings within individuals; results are summarized in a Manhattan plot showing associations between embeddings and genetic variants. **c** Visualization of histological changes associated with significant variants (e.g., *rs7030256*) by projecting embeddings—interpolated along the genetic effect direction—back into image space using the generative decoder

Here, we introduce HistoGWAS, an AI-enabled framework for genome-wide association studies (GWAS) of histological traits. The approach begins with a semantic autoencoding strategy that leverages pretrained foundation models to derive quantitative histology embeddings for genetic analysis, and a generative decoder to invert the encoding process (Fig. 1a). We then perform scalable GWAS of these embeddings using an efficient variance component testing framework (Fig. 1b). Finally, the generative decoder is used to visualize tissue features associated with significant genetic loci (Fig. 1c).

Notably, HistoGWAS enables the identification of genome-wide significant loci influencing tissue-level features from histological data—genetic associations we term tissueQTLs. Simulations further show increased detection power with larger sample sizes, supporting its applicability to biobank-scale genetic studies of tissue phenotypes.

## Results

### Semantic autoencoding of histology for genetic analysis

**Fig. 2:**
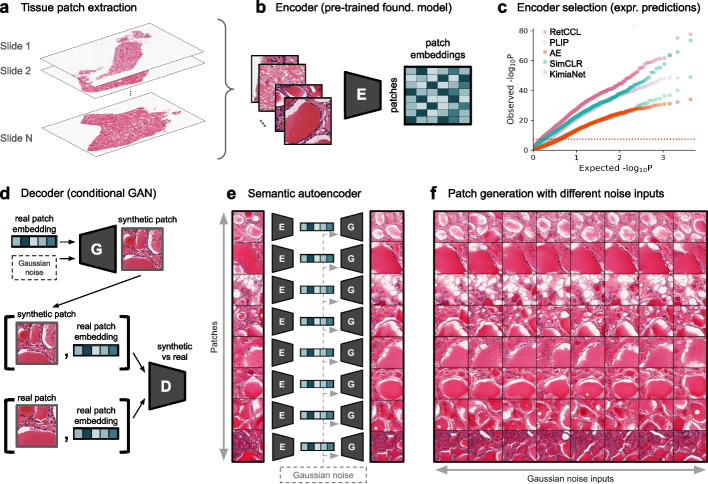
HistoGWAS semantic autoencoding framework. **a** Tissue patches (192 μm x 192 μm) are extracted from 9,006 whole-slide images, yielding 22,812,099 patches after quality control (Methods; Additional file 1: Dataset S1). **b** Patch-level embeddings are derived using several pretrained models as candidate semantic encoders: RetCCL [21], SimCLR [22, 36], KimiaNet [37], PLIP [21], and reconstruction-based autoencoders [30, 32]. **c** Model comparison for gene expression prediction in thyroid tissue, shown as a quantile–quantile (QQ) plot of per-gene -log_10_ P values from association tests between observed and predicted expression levels in test-set individuals (Methods). Each point represents a gene. The top-performing model is selected as the encoder. **d** Conditional generative adversarial network (GAN) framework in which a generator (G) produces synthetic patches from embeddings and Gaussian noise, while a discriminator (D) distinguishes real from generated images conditional on embeddings. **e** The trained generator functions as a decoder, reconstructing semantically similar patches from embeddings. **f** Reconstructions for different embeddings (rows) and Gaussian noise samples (columns). Varying noise at fixed embedding yields distinct reconstructions that preserve key structural features

First, we developed an autoencoding strategy in which a pre-trained encoder is selected to maximize molecular prediction performance and a decoder is trained to invert the encoding process. We analyzed histology samples from the Genotype-Tissue Expression (GTEx) dataset [2], focusing on eleven tissues with the highest availability of both histology and genetic data (n ≥ 750; Additional file 1: Dataset S1). Following previous studies [22, 35], we extracted 192 μm x 192 μm tissue patches from each whole-slide image (Fig. 2a), yielding 22,812,099 patches across 9,006 slides after rigorous quality control (Methods; Additional file 1: Dataset S1).

Starting with the encoder, we identified the pretrained model whose slide embeddings best linearly predict gene expression (Fig. 2b; Methods). We compared four types of pretrained models: (i) self-supervised contrastive learning methods that learn generic tissue features without labels (SimCLR [36] and RetCCL [22]); (ii) a supervised cancer type classification model (KemiaNet [37]); (iii) a cross-modal foundation model aligning histology with pathologist text descriptions (PLIP [21]); and (iv) standard autoencoders optimized for image reconstruction (AE) [30, 32]. Contrastive learning methods performed best, with RetCCL achieving the highest average Spearman correlation across genes (e.g., in thyroid tissue: ρ = 0.366 ± 0.008 for RetCCL, ρ = 0.346 ± 0.008 for SimCLR, ρ = 0.310 ± 0.008 for KemiaNet, ρ = 0.270 ± 0.010 for PLIP, and ρ = 0.230 ± 0.007 for AE; Fig. 2c; Additional file 2: Fig. S1-S2). RetCCL also predicted the largest number of genes above multiple correlation thresholds (e.g., in thyroid tissue: RetCCL predicted 777 genes with ρ > 0.5, 18.33% of total; SimCLR 613 [14.46%]; KemiaNet 131 [3.09%]; PLIP 254 [5.99%]; and 111 [2.62%] for AE; Additional file 2: Fig. S2). Based on these results, we selected RetCCL as the HistoGWAS encoder.

After selecting the encoder, we developed a decoder to reconstruct full-resolution images from patch embeddings. To this end, we trained conditional generative adversarial networks (GANs) [38–40] for each tissue, conditioning image generation on encoder-derived embeddings. Our GAN framework comprised a generator that produces synthetic patches from embeddings and Gaussian noise, and a discriminator trained to distinguish real from generated images conditional on those embeddings (Fig. 2d, Methods). After adversarial training, the generator produced realistic images conditional on the embeddings, serving as a decoder (Fig. 2e). Reconstructions from this decoder are stochastic: fixing the embedding while varying the Gaussian noise yields distinct images that retain key features of the original patch (Fig. 2f).

Embeddings from images reconstructed with the HistoGWAS semantic autoencoder remained highly predictive of gene expression (Additional file 2: Fig. S3). To quantify information loss, we compared prediction performance using embeddings from original and reconstructed images as inputs to a model predicting gene expression, testing for each gene whether performance was significantly reduced (Methods). In thyroid tissue, only 326 genes (7.7% of total, Bonferroni-adjusted *P* < 0.05) showed significant deterioration with HistoGWAS reconstructions, compared to 1,496 genes (35.3%) with the standard autoencoder, confirming that HistoGWAS retains substantially more predictive signal for gene expression levels (Additional file 2: Fig. S3).

### Genome-wide association analysis across 11 tissues identifies four tissueQTLs

To capture subtle genetic influences on tissue subregions, we focused our genetic analyses on 68 distinct cluster signatures identified through refined clustering within each tissue (Methods; Additional file 2: Fig. S4).

First, to validate the biological relevance of these cluster signatures, we correlated slide-level cluster proportions with bulk gene expression across individuals. This analysis identified distinct genes and pathways associated with different signatures within the same tissue, highlighting their functional diversity (Methods; Additional file 2: Fig. S5; Additional file 3: Dataset S2).

**Fig. 3:**
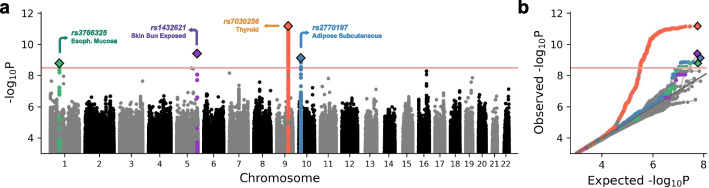
GWAS of histological embeddings across 68 cluster signatures identifies four tissueQTLs. **a** Manhattan plot showing genome-wide association results across 68 cluster signatures. Red horizontal lines indicate multi-trait genome-wide significance thresholds corresponding to a 20% family-wise error rate (FWER), determined through a permutation-based procedure (Methods). Lead variants at significant tissueQTLs are marked with diamonds; variants in linkage disequilibrium (R.^2^ > 0.5) with the lead are color-coded accordingly. **b** QQ plots of association -log_10_ P values stratified by tissue type, with variants color-coded as in (**a**)

Next, we employed an adapted scalable variance component model to test genome-wide associations between individual-level average patch embeddings for each cluster signature and each of ~ 5 million variants with minor allele frequency (MAF) ≥ 5%, adjusting for patient covariates and population structure (Methods). Across 68 signatures, we identified four genome-wide significant tissueQTLs at 20% FWER (P < 3.23 × 10^–9^; Fig. 3a, b; Additional file 2: Fig. S6; Additional file 4: Dataset S3), with the strongest signal also exceeding the 5% FWER threshold (P < 4.29 × 10^–10^). Analysis of permuted genotypes yielded calibrated P values with no significant associations (Additional file 2: Fig. S7), supporting proper calibration of our testing procedure. In contrast, embeddings from standard autoencoders identified only a single locus at 20% FWER, corresponding to the same top signal detected by HistoGWAS (Additional file 2: Fig. S8).

The most significant tissueQTL, lead intronic variant *rs7030256* in the *PTCSC2* gene (P < 6.62 × 10^–12^), was associated with five different signature clusters in thyroid tissue (Additional file 2: Fig. S6). The other three tissueQTLs were each associated with a single cluster signature in sun-exposed skin (*rs1432621*; P < 3.94 × 10^–10^), adipose subcutaneous (*rs2770197*; P < 7.39 × 10^–10^), and esophagus mucosa (*rs3766325*; P < 1.64 × 10^–9^), respectively.

### Linking tissueQTLs to molecular and disease traits

To interpret the identified tissueQTLs, we implemented downstream analyses linking genetic associations to molecular traits, disease risk, and histological phenotypes.

**Fig. 4:**
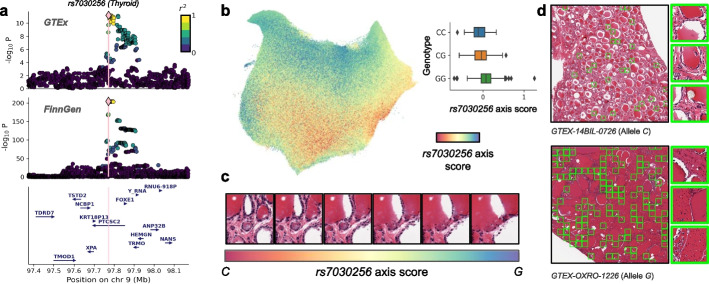
Effect of tissueQTL *rs7030256* on thyroid histology. **a** Dual LocusZoom plots showing colocalization for *rs7030256* (PP-H4 = 0.97): the upper panel displays association with the GTEx thyroid cluster signature 2, and the lower panel shows the FinnGen GWAS signal for autoimmune hypothyroidism [44]. Nearby genes are annotated for genomic context. **b** UMAP of thyroid patch embeddings colored by *rs7030256* axis scores (Methods), with a box plot showing score distributions across genotypes. **c** Histological images illustrating allele effects by projecting interpolated embeddings—along the *rs7030256* genetic effect axis—back into image space using the semantic decoder. **d** Representative whole-slide images from samples with strong phenotypic signals along the *rs7030256* genetic effect axis (Methods). Patches in the top and bottom 5% of axis scores are highlighted in green, with three regions per slide magnified to illustrate consistent morphological differences. Additional examples are shown in Additional file 2: Fig. S11

We performed formal colocalization analyses using established frameworks [41], leveraging outputs from our variance component model (Methods). For each of the four genome-wide significant tissueQTLs, we tested for shared genetic signals with disease GWAS loci, expression QTLs (eQTLs), and splicing QTLs (sQTLs). The top thyroid tissueQTL (*rs7030256*) colocalized with hypothyroidism and goiter in FinnGen [42–44] (PP-H4 = 0.97; Fig. 4a) and with *FOXE1* eQTLs in GTEx thyroid (PP-H4 = 0.94; Additional file 2: Fig. S9). The *G* allele was associated with increased disease risk and higher *FOXE1* expression, consistent with its role as a master regulator of thyroid development and an established cancer susceptibility locus [45]. Additional examples included skin tissueQTL *rs1432621* colocalizing with FinnGen “Other arthrosis” [46] (PP-H4 = 0.79; allele *A* increasing risk), esophagus tissueQTL *rs3766325* with *IFI44* eQTLs (PP-H4 = 0.98; allele *G* increasing expression), and adipose tissueQTL *rs2770197* with *ST8SIA6* sQTLs (PP-H4 = 0.99; Additional file 2: Fig. S9).

Next, to visualize predicted histological effects of tissueQTLs, we applied latent-space interpolation using the HistoGWAS decoder. For each tissueQTL lead variant, we defined a genetic effect axis in embedding space and generated images from interpolated embeddings along this axis (Methods), enabling direct visualization of histological changes associated with the genetic variant under study. Using *rs7030256* in thyroid as an example, pathologist assessment of generated images revealed progressive follicular enlargement and colloid accumulation with the *G* allele, often accompanied by inflammatory infiltrates (Fig. 4b, c; Additional file 2: Fig. S10), consistent with goiter development. In contrast, the *C* allele was associated with a white rim around the colloid, suggestive of increased resorption and thyroid activity (Fig. 4b, c; Additional file 2: Fig. S10). To contextualize these effects, we examined whole-slide images from samples with strong phenotypic signals along the inferred genetic axis (Methods), confirming consistent differences in follicular architecture and colloid content (Fig. 4d; Additional file 2: Fig. S11). Other loci also showed interpretable patterns: *rs3766325* in esophagus mucosa was associated with enlarged epithelial nuclei indicative of regeneration; *rs1432621* in skin with increased collagen density; and *rs2770197* in adipose with enlarged vacuoles and cell membrane deterioration (Additional file 2: Figs. S10, S12–S14).

Finally, to link tissueQTLs to molecular functions, we performed transcriptome-wide association analyses by testing each tissueQTL lead variant for association with the expression of every gene individually, followed by pathway enrichment (Methods). For *rs7030256* in thyroid, we identified 128 significantly associated genes (Bonferroni-adjusted P < 0.05; Additional file 4: Dataset S3), with enrichment in hedgehog signaling and estrogen response pathways. Other loci also showed molecular signatures: *rs3766325* in esophagus was associated with 30 genes (Bonferroni-adjusted P < 0.05); genes associated with *rs1432621* in skin were enriched for UV response and epithelial–mesenchymal transition pathways [47–49]; and genes associated with *rs2770197* in adipose were enriched for adipogenesis and fatty acid metabolism pathways [19, 50] (Additional file 4: Dataset S3).

Together, these analyses link tissueQTLs to molecular functions, disease associations, and histological phenotypes, providing a framework for evaluating their biological relevance.

### Power analysis of HistoGWAS

To evaluate the statistical power of HistoGWAS, we conducted simulations of cohorts with up to 10,000 individuals. Trait embeddings were generated as functions of covariates, a genetic variant, and Gaussian noise (Methods). After confirming proper calibration under the null (no genetic effect; Additional file 2: Fig. S15), we estimated power to detect tissueQTLs at genome-wide significance (*P* < 5 × 10⁻⁸) across varying sample sizes and effect sizes.

**Fig. 5:**
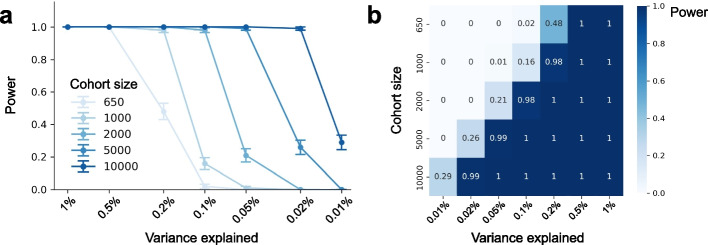
Power analysis of HistoGWAS across sample sizes and effect sizes. **a** Statistical power to detect significant tissueQTLs (*P* < 5 × 10⁻⁸) across combinations of variance explained and cohort size. Standard errors are based on 100 simulation seeds. **b** Heatmap showing average power for each combination of cohort size (columns) and variance explained (rows), providing guidance for tissueQTL study design

In sample sizes comparable to our study (650–1,000 individuals), HistoGWAS achieved high power to detect variants explaining at least 0.2% of variance in the embedding space, but had limited power for effects explaining ≤ 0.1% variance (Fig. 5a). Power increased substantially with larger cohorts, enabling detection of variants explaining ≥ 0.1%, ≥ 0.05%, and ≥ 0.02% of variance with 2,000, 5,000, and 10,000 individuals, respectively. Figure 5b summarizes the sample sizes required to achieve target power across effect sizes, providing guidance for the design of future genome-wide association studies of histology phenotypes.

## Discussion

HistoGWAS establishes a unified pipeline for tissueQTL mapping by integrating pretrained histology models for automated phenotyping, variance component tests for scalable genetic association, and generative models for interpretability. Applied to GTEx histology and genetic data, the framework identified four significant tissueQTLs, providing a proof of principle for linking tissue morphology to molecular and disease traits. Among these, the *PTCSC2* locus in thyroid—a lncRNA gene implicated in thyroid biology [51–53]—was associated with changes in follicle size and colloid content and colocalized with both a thyroid disease risk locus [44] and a *FOXE1* eQTL, implicating a master regulator of thyroid development.

Despite its advantages, HistoGWAS is not without limitations. Our focus on tissue-specific cluster signatures enables high-throughput discovery and interpretable visualization of variant effects, but defining such signatures may be challenging in highly heterogeneous tissues—such as tumors or complex microenvironments—or when genetic effects span multiple spatial scales. Patch size is a key parameter that determines analytical resolution: smaller patches emphasize cellular detail (as in this study), whereas larger patches capture broader tissue architecture. Whole-slide representation learning and multi-resolution approaches offer promising strategies to preserve spatial context while capturing heterogeneity across scales [54–56].

A second limitation concerns the generative decoder, which is currently trained separately for each tissue. While this design yields accurate reconstructions, it requires training multiple decoders. Preliminary results on cross-tissue training with learnable tissue embeddings are promising (Additional file 2: Fig. S16), but further refinement and systematic evaluation are needed. Conditional diffusion models may provide a more flexible framework for histological reconstruction [57].

Third, although HistoGWAS can in principle be applied to rare variants, our analysis was restricted to common variants due to limited power for testing single rare variants at GTEx sample sizes. Future studies could leverage gene-level rare variant tests that incorporate functional annotations to improve power [58–61].

Finally, although we selected the encoder based on gene expression prediction to obtain general-purpose embeddings capturing broad tissue processes, alternative evaluation or fine-tuning strategies—such as optimizing for specific clinical labels—may be preferable in other settings.

## Conclusions

We introduced HistoGWAS as a scalable framework for systematic discovery of tissueQTLs from histology. By combining pretrained encoders, variance component models, and generative decoders, it links genetic variants to tissue features, extending genetic analyses of intermediate phenotypes to histology.

The integration of AI-based encoding and decoding of high-content phenotypes with multivariate analysis of latent representations is applicable beyond histology. Similar strategies could facilitate the study of genetic effects on morphological variation in non-invasive medical imaging [8–14, 62] and the evaluation of perturbation effects in high-content screens [63]. Such extensions may be particularly relevant for advanced in vitro models, including organoids, where genetic or chemical perturbations can be linked to complex in vitro phenotypes.

Power analysis indicates that HistoGWAS is suitable for large-scale histology cohorts, enabling detection of genetic effects on tissue structure. By linking germline variation to histological features, the framework supports genetically informed investigation of disease mechanisms [64, 65]. Together, these elements establish HistoGWAS as a foundation for tissueQTL mapping and future genetic studies of tissue phenotypes.

## Methods

### Study aim and design

The aim of this study was to develop HistoGWAS, a scalable AI-enabled framework for genome-wide association studies of histological phenotypes. The framework integrates three components: (i) semantic autoencoding of histology images to derive quantitative tissue embeddings, (ii) variance component models for association testing between genetic variants and embeddings, and (iii) generative modeling to reconstruct and interpret the histological effects of genetic variation. For validation, we applied HistoGWAS to GTEx, selecting tissues with both histology and germline genetic data available for at least 750 individuals. This yielded a dataset comprising 11 tissue types and 9,006 hematoxylin and eosin (H&E)–stained slides, corresponding to 22.8 million image patches (Additional file 1: Dataset S1). The study was structured as a computational pipeline consisting of preprocessing, encoder selection, generative decoder training, genetic association analysis, and downstream characterization of significant loci. The following sections describe each component in detail, together with the power analysis.

### Preprocessing and encoder selection

#### Data preprocessing and patch extraction

We curated an initial dataset from GTEx, selecting tissues with both histological and genetic data available for at least 750 individuals. This resulted in 11 tissue types and 9,006 slides (Additional file 1: Dataset S1). For each slide, we extracted 192 μm × 192 μm patches using a predefined grid. This scale captures both cellular detail and local tissue architecture and matches the resolution used in recent histology foundation models [21, 22]. Slides were converted to grayscale, and tissue regions were identified using binary thresholding (cv2.threshold, *OpenCV* [66]) to distinguish foreground from background. Patches containing at least 50% tissue were retained and exported as 256 × 256 pixel images (0.75 μm per pixel), yielding 22,812,099 patches across 11 tissues (Additional file 1: Dataset S1).

#### Compared models for semantic encoding

We evaluated four pretrained models as candidate semantic encoders for HistoGWAS: (i) RetCCL [21], pretrained on The Cancer Genome Atlas (TCGA) using cluster-guided contrastive learning; (ii) SimCLR [22, 36], pretrained on ImageNet with a standard contrastive learning procedure; (iii) KimiaNet [37], pretrained on TCGA for cancer type classification; and (iv) PLIP [21], pretrained on slide images and associated pathologist descriptions from OpenPath using a multimodal contrastive-learning framework. These models were pretrained on large, heterogeneous datasets with extensive augmentations (e.g., color jittering, blurring, rotations), promoting robustness to moderate image-quality variation. All were applied to GTEx without fine-tuning. In addition, we trained a classical autoencoder (AE) separately for each of the 11 tissues using an L2 reconstruction loss (Additional file 2: Section S1.1), as in prior histology–genomics analyses of GTEx [30, 32]. After computing embeddings for all foreground patches, we performed Principal Component Analysis (PCA) within each tissue and retained the top 64 principal components. The number of components was selected based on simulations assessing calibration of genome-wide association P values under null model simulations (Additional file 2: Fig. S17).

#### Encoder performance evaluation and selection

We evaluated five candidate models by assessing how well their embeddings predicted gene expression across the 11 tissues. For each tissue, individual-level embeddings were computed by averaging patch-level embeddings per individual. For each gene, we fitted a variance component model with expression as the outcome (log₁₀ TPM; GTEx V8 [67]) and embedding effects modeled as random effects, focusing on highly variable genes identified using scanpy’s highly_variable_genes function [68]. Models were trained on 50% of individuals and evaluated on the remaining 50% using leave-one-out best linear unbiased prediction [69, 70]. Prediction accuracy was quantified by Spearman’s correlation between observed and predicted expression in the test set, with significance defined by Bonferroni-corrected *P* < 0.05. In addition to mean pooling, we benchmarked attention-based pooling using the MixMIL framework [71]. Across models and pooling strategies, RetCCL achieved the highest predictive accuracy and was selected for subsequent HistoGWAS analyses (Fig. 2c; Additional file 2: Fig. S1-2).

### Generative decoder

To invert the encoding process, we trained a generator within a conditional generative adversarial network (cGAN) framework [38], adapted to condition on patch embeddings from the HistoGWAS encoder. The generator produces synthetic patch images from embeddings, while the discriminator distinguishes real from generated images conditional on those embeddings. To generate high-resolution (256 × 256) images, we adopted the progressive training strategy of Progressive GANs [40], incrementally adding layers to the generator and discriminator to capture increasingly fine-grained detail during training [40].

#### Architecture of the generator network

The generator maps a 512-dimensional latent representation $\boldsymbol{z}$ to an image via a convolutional function $G(\boldsymbol{z})$. In contrast to classical GANs, where $\boldsymbol{z}\sim \mathcal{N}(\boldsymbol{0}, \boldsymbol{I})$, we condition $\mathbf{z}$ on 64-dimensional patch embeddings $\boldsymbol{x}$ by modeling it as a multivariate normal with mean ${m}_{z}(\boldsymbol{x})$ and standard deviation ${s}_{z}(\boldsymbol{x})$, both parameterized as functions of $\boldsymbol{x}$. Using the reparameterization trick [74], we express $\boldsymbol{z}=m_z\left(\boldsymbol{x}\right)+s_z\left(\boldsymbol{x}\right)\odot\boldsymbol{\epsilon}$, with $\boldsymbol{\epsilon} \sim N(\boldsymbol{0}, \boldsymbol{I})$ and $\odot$ denoting the Hadamard product, separating conditioning from stochasticity and thus enabling gradient-based optimization. Both $m_z(\boldsymbol{x})$ and $s_z(\boldsymbol{x})$ are implemented as linear layers, with a softplus activation applied to $s_z(\boldsymbol{x})$ to ensure non-negativity. The overall architecture adapts the publicly available Progressive GAN implementation in [72]; detailed specifications are provided in the referenced code base.

#### Architecture of the discriminator module

Following [40], the discriminator processes a patch image $\boldsymbol{y}$ to produce a 512-dimensional representation $\boldsymbol{h}=D(\boldsymbol{y})$. The scalar output for each patch is computed as the sum of an unconditional component (a linear layer applied to $\boldsymbol{h}$) and a conditional component. The conditional term is defined as the dot product between $\boldsymbol{h}$ and a 512-dimensional encoding of the observed patch embedding, obtained via a linear transformation of the embedding. This conditioning strategy follows BigGAN [73], where class-specific learnable embeddings are used; here, we instead condition on a learnable linear transformation of the observed patch embeddings. Detailed architectural specifications are provided in the original implementation [72].

#### Progressive training and optimization details

We trained the model using the Wasserstein GAN loss [74] with gradient penalty (λ = 10) to improve stability and convergence. Following [72], training began at 4 × 4 resolution and progressively increased through 8 × 8, 16 × 16, 32 × 32, 64 × 64, 128 × 128, and finally 256 × 256 pixels by incrementally adding layers to both the generator and discriminator. The initial resolution was trained for 48,000 iterations, and each subsequent stage for 96,000 iterations, with a batch size of 64 patches. We used the Adam optimizer [75] with β₁ = 0, β₂ = 0.99, and a learning rate of 0.01, updating generator and discriminator iteratively to balance training dynamics [76].

#### Validation of semantic reconstruction accuracy

The semantic autoencoder combines RetCCL with dimensionality reduction to 64 principal components for encoding and the generator of the conditional GAN for decoding. After qualitative assessment of original and reconstructed patches under varying noise inputs (Fig. 2e, f), we performed quantitative validation using gene expression prediction. For each tissue, a variance component model was trained on 50% of individuals using embeddings from original patches. The trained model was then applied to the remaining 50%, using embeddings derived either from original or reconstructed patches. For each gene, we tested whether the correlation between predicted and observed expression using reconstructed embeddings was significantly weaker than that using original embeddings, applying Steiger’s z-test for dependent correlations [77] (*cocor* package [78]). Significant deterioration was defined as Bonferroni-adjusted *P* < 0.05 (correction across genes). The analysis was conducted separately for the HistoGWAS semantic autoencoder and a conventional autoencoder baseline, demonstrating improved preservation of predictive signal with HistoGWAS (Additional file 2: Fig. S3). Detailed procedures are provided in Additional file 2: Section S1.2.

### Genetic analysis

#### Definition of histological cluster signatures for GWAS

To capture diverse histological phenotypes within each tissue, we performed unsupervised analysis of RetCCL embeddings using scanpy [68]. For each tissue, embeddings were reduced to the top 64 principal components via PCA, followed by construction of a nearest-neighbor graph (n_neighbors = 10), Uniform Manifold Approximation and Projection (UMAP) [79], and Leiden clustering (resolution = 0.5) [80]. Hyperparameters were selected to identify large, morphologically homogeneous clusters (Additional file 2: Figs. S4–S5). To ensure sufficient representation for genetic analysis, we retained clusters represented by at least 10 patches per slide in at least 650 slides. This yielded 68 tissue signature clusters comprising 19,901,526 patches (Additional file 2: Figs. S4–S5). For biological interpretation, we generated cluster prototypes by conditioning the generative decoder on centroid embeddings and sampling multiple noise realizations (8 images per cluster). These, together with representative real images at multiple magnifications, were reviewed and annotated by a medical researcher with formal histology training (Additional file 5: Dataset S4). Across tissues, the number of individuals included in GWAS ranged from 650 to 815 (mean 748; Additional file 6: Dataset S5).

#### Associating cluster signatures with gene expression

To validate the histological cluster signatures, we examined their association with gene expression. For each signature, slide-level abundance was defined as the proportion of patches assigned to that signature and correlated across slides with expression of each gene using a univariate linear model. Gene expression values were Gaussianized and modeled as the dependent variable without additional covariates. Significance was assessed using a log-likelihood ratio test. QQ plots for each signature, highlighting the top five associated genes and representative patches, are shown in Additional file 2: Fig. S5. Full results are provided in Additional file 3: Dataset S2.

#### Association testing framework

We employed a variance component test within a linear mixed model framework to assess genetic associations with histological traits. This approach enables multivariate testing of high-dimensional embeddings against single genetic variants. Given the genotype vector $\boldsymbol{g}$ for a variant across $N$ individuals, the $N\times L$ matrix of individual-level embeddings $\boldsymbol{X}$, and the $N\times K$ covariate matrix $\boldsymbol{F}$, we considered the generalized variance component model:

$$\mathrm{link}^{-1}\left(\boldsymbol {g}\right)=\boldsymbol{F}\boldsymbol{\alpha}+\boldsymbol{u},\;\mathrm{where}\;\boldsymbol u\;\sim\;\mathcal N\left(\boldsymbol{0},\;\sigma_x^2\;\boldsymbol K\left(\boldsymbol X\right)\right)$$

Here, the link function depends on the assumed likelihood for genotype values, **α** denotes covariate effects, and $\boldsymbol{K}(\boldsymbol{X})$ is an $N\times N$ cosine similarity-based covariance matrix [81] capturing pairwise similarity between individuals based on embeddings. Association was tested via a variance component score test of $\sigma_x^2>0$ [82], analogous to sequence kernel association tests [83, 84], with P values computed using the Davies method [85] or the Liu saddlepoint approximation when required [86]. We evaluated both binomial [71, 87] and Gaussian likelihoods for genotype modeling. While both were well calibrated, we selected the Gaussian formulation for its superior computational efficiency. Computational efficiency was achieved by exploiting the low-rank structure of the embedding covariance [84, 88–91]. Within this framework, we tested associations between 68 cluster signature embeddings and approximately 5 million common variants (MAF ≥ 5%), adjusting for sex, age, type of death, and the first four genetic principal components. Further methodological details are provided in Additional file 2: Section S1.3.

#### Multiple hypothesis testing correction

To account for multiple hypothesis testing, we employed a permutation-based procedure. For each of the 68 cluster signatures, we performed 100 genotype permutations, yielding 6,800 genome-wide association analyses under the null. For each permutation, the minimum P value across all variants was recorded, producing a null distribution of 6,800 minimum P values. The empirical 20% FWER threshold (α = 0.2) was defined as the 20th percentile of this distribution. To further account for testing across 68 cluster signatures, this threshold was divided by the number of clusters, resulting in a final significance threshold of P < 3.23 × 10^–9^.

### Downstream analyses

#### Colocalization

To assess whether lead variants shared causal signals with known molecular or complex traits, we derived approximate variant-level Bayes factors from the HistoGWAS variance component model, compatible with *coloc* (via coloc.bf_bf) [41]. This enabled formal colocalization analyses with external summary statistics from disease GWAS and molecular QTL studies. Bayes factors were approximated using the Bayesian Information Criterion (BIC) [92], assuming one degree of freedom corresponding to the variance component parameter. Likelihood ratio statistics were estimated asymptotically from chi-square statistics derived from score test P values [93]. For each tissueQTL, we applied *coloc* to test colocalization with (i) phenome-wide significant FinnGen traits (FinnGen browser) and (ii) all cis-eQTLs and cis-sQTLs across tissues from the GTEx Portal. Analyses were performed in 1 Mb windows centered on each tissueQTL using default *coloc* parameters.

#### Visualization of genetic effects on histology

To visualize histological effects of significant variants, we combined embedding interpolation with semantic decoding. For each variant, we first estimated its direction of effect in embedding space using the variance component model, defining a “genetic effect axis.” We then computed representative extreme embeddings by averaging patch-level embeddings within the 1st–5th and 95th–99th percentiles of projection scores along this axis. Interpolating between the representative extreme embeddings and projecting the obtained interpolations back into the image space via the semantic decoder enabled visualization of histological variation along the genetic effect axis. Given the stochastic nature of the decoder, multiple visual realizations can be generated by varying the input Gaussian noise (Additional file 2: Fig. S10). Full details of this procedure are provided in Additional file 2: Section S1.4. To contextualize the generated histological changes, we additionally visualized whole-slide images exhibiting strong phenotypic signals along the genetic effect axis, selecting slides with at least 40 patches in the top or bottom 5% of projection scores and highlighting these patches. This approach provides genotype-independent visualization of extreme phenotypes in their native tissue context.

#### Molecular and complex trait associations of tissueQTLs

We evaluated whether tissueQTLs are associated with changes in gene expression, pathway activity, or complex traits. Associations with gene expression were tested between the tissueQTL lead variant and Gaussianized expression of each highly variable gene in the same tissue, adjusting for sex, age, type of death, and the first four genetic principal components; significance was defined at Bonferroni-adjusted *P* < 0.05. Pathway enrichment was assessed using Fisher’s exact test via the enrichr function in *gseapy* [94], based on *MSigDB_Hallmark_2020* annotations [95], focusing on the top 50 positively and negatively associated genes. The five pathways showing the strongest enrichment were selected for further interpretation. Additionally, the Open Targets Genetics platform [96] was queried to explore associations with complex traits.

### Power analysis

To evaluate statistical power under diverse scenarios, we simulated individual-level embeddings as additive effects of covariates (sex, age, and genetic principal components), a genetic variant, and Gaussian noise. Associations between simulated embeddings and genetic variants were tested using the HistoGWAS framework, with power evaluated at genome-wide significance threshold (*P* < 5 × 10^–8^). Power was estimated across cohort sizes (650, 1,000, 2,000, 5,000, 10,000) and genetic effect sizes explaining 0.01%, 0.02%, 0.05%, 0.1%, 0.2%, 0.5%, 1% of variance, using 100 simulation seeds per scenario. Detailed simulation procedures are described in Additional file 2: Section S1.5.

## Supplementary Information

Additional file 1: Dataset S1. Overview of genotype, expression, and histology data across the analyzed GTEx tissues.

Additional file 2: Supplementary information. Detailed methodological descriptions and Figs S1–S17.

Additional file 3: Dataset S2. Association statistics linking cluster signature abundance to gene expression levels across tissues.

Additional file 4: Dataset S3. Association statistics for lead variants at genome-wide significant loci, including links to expression of highly variable genes and pathway enrichment results for upregulated and downregulated gene sets.

Additional file 5: Dataset S4. Histological annotations for all 68 cluster signatures described in the Methods.

Additional file 6: Dataset S5. Sample sizes used in GWAS analyses for each histological cluster signature.

## Data Availability

The Python implementation of HistoGWAS, including modules for data preprocessing, encoder selection, generative decoder training, genetic association testing, and downstream locus characterization, is available at [https://github.com/AIH-SGML/HistoGWAS] [97] (BSD license). This study uses data from the GTEx Project (V8 release; dbGaP accession phs000424.v8.p2). Publicly available processed gene expression matrices and covariates were obtained from [https://gtexportal.org/home/datasets], and histological whole-slide images from [https://www.gtexportal.org/home/histologyPage]. The histology embeddings generated in this study have been deposited in Zenodo and are publicly available at [https://zenodo.org/records/18773562] [98]. Controlled-access whole genome sequencing (WGS) phased genotype data were accessed through the AnVIL repository under approved dbGaP application number #32009 (see [https://gtexportal.org/home/protectedDataAccess]). No controlled-access data are redistributed in this study. External software, datasets, and pretrained models used in this work include: • RetCCL (commit a85b972): [https://github.com/Xiyue-Wang/RetCCL] • SimCLR implementation (ImageNet pretrained checkpoint): [https://pl-bolts-weights.s3.us-east-2.amazonaws.com/simclr/bolts_simclr_imagenet/simclr_imagenet.ckpt] • KimiaNet (commit 4a106ac): [https://github.com/KimiaLabMayo/KimiaNet] • PLIP (commit f010f3d): [https://github.com/PathologyFoundation/plip] • coloc R package (version 5.2.3): [https://cran.r-project.org/package=coloc] • FinnGen summary statistics (release 10): [https://finngen.gitbook.io/documentation/] • GTEx v10 cis-eQTL and cis-sQTL summary statistics used for colocalization analyses: [https://gtexportal.org/home/downloads/adult-gtex/qtl]
