# Supplementary material for: HistoGWAS: an AI-enabled framework for automated genetic analysis of tissue phenotypes in histology cohorts
Source: Genome Biol. 2026 Mar 31;27:122. doi: 10.1186/s13059-026-04031-z (PMC13063728; doi:10.1186/s13059-026-04031-z)
Supplement: Supplementary file 2 — Additional file 2: Supplementary information. Detailed methodological descriptions and Figs S1–S17. [file 13059_2026_4031_MOESM2_ESM.pdf]

# Supplementary information for “HistoGWAS: an AI framework for automated and interpretable genetic analysis of tissue phenotypes”

## Contents

**S1 Supplementary Methods** **1**
S1.1 Standard Autoencoder . . . . . . . . . . . . . . . . . . . . . . . . . . . . . 1
S1.2 Procedure for Validating the HistoGWAS Semantic Autoencoder using Expression Data . . . . . . . . . . . . . . . . . . . . . . . . . . . . . . . . 2
S1.3 Association Testing Framework . . . . . . . . . . . . . . . . . . . . . . . . 3
S1.4 Visualization of Genetic Effects on Histology . . . . . . . . . . . . . . . 4
S1.5 Power Analysis for HistoGWAS . . . . . . . . . . . . . . . . . . . . . . . 6

**S2 Supplementary Figures** **7**

## S1 Supplementary Methods

### S1.1 Standard Autoencoder

In our analyses, we compared the HistoGWAS semantic autoencoder with a standard autoencoder optimized for image reconstruction, which we implemented following previous work leveraging the GTEx resource for molecular analyses. We considered the same architecture and optimization procedure as implemented in [1], optimizing the mean square loss between the reconstructed and original images using the Adam optimizer with learning rate 1e-4. Full details on its architecture and optimization details can be found below.

**Architecture of encoder.** The standard autoencoder consists of two primary components: encoder and decoder. The encoder consists of a series of five convolutional layers, each followed by a max-pooling layer and ReLU activation. The convolutional layers incrementally increase the number of channels from 3 to 128 (3, 16, 32, 64, 128),

maintaining a constant kernel size of 3 and stride of 1 with padding. Following the convolutional layers, a flattening step transitions the data from a 3D tensor to a 1D tensor followed by a linear layer to produce the encoded representation of dimension 1024.

**Architecture of decoder.** The decoder reconstructs the input image from encoded the representation. Starting with a linear layer that expands the representation back to spatial dimensions (128 channels and 8×8 spatial size), then a series of upsample operations followed by convolutional layers and ReLU activation. Through these operations, the channels incrementally decrease from 128 to 3 (128, 64, 32, 16, 3). We exceptionally use the Tanh activation (rather than ReLu) after the last convolutional layer.

## S1.2 Procedure for Validating the HistoGWAS Semantic Autoencoder using Expression Data

This section outlines the step-by-step procedure used to quantitatively validate the semantic autoencoder reconstructions using gene expression measurements.

1. **Computation of Individual-Level Embeddings from Real Patches**
   - Extract patch-level embeddings from real histological patches using a pre-trained encoder (e.g., RetCCL);
   - Train and apply Principal Component Analysis (PCA) to reduce the dimensionality of the embeddings, retaining 64 components based on calibration analysis (**Supplementary Figure 17**);
   - Average the reduced patch-level embeddings across all patches from the same individual to obtain individual-level embeddings.
2. **Computation of Individual-Level Embeddings from Reconstructions**
   - Start from the reduced patch-level embeddings obtained in Step 1;
   - Generate reconstructed patches from these embeddings using the semantic decoder;
   - Re-encode the reconstructed patches using the same encoder as in Step 1;
   - Apply the previously trained PCA transformation to the re-encoded embeddings;
   - Aggregate the PCA-reduced patch-level embeddings across patches from each individual to derive individual-level embeddings.
3. **Model Training and Out-of-Sample Evaluation**
   - For each gene:
     a) Train a linear mixed model using 50% of the individuals to predict $\log_{10}$ TPM expression values from individual-level embeddings derived from real patches. The model includes a fixed intercept and random effects corresponding to the embeddings;
     b) Use the resulting model to generate out-of-sample predictions on the remaining 50% of individuals using Best Linear Unbiased Predictions (BLUP) [7], with embeddings derived either from real or reconstructed patches;

- To assess reconstruction-induced performance loss, we applied Steiger's Z test for dependent correlations (as implemented in the `cocor` package [5]). For each gene, we computed three Pearson correlations from subject-level data: (i) observed expression vs. predictions from real patches, (ii) observed expression vs. predictions from reconstructed patches, and (iii) predictions from real vs. predictions from reconstructed patches. These three correlations were used to test whether predictive performance significantly deteriorated when using reconstructed patches. We used the one-sided form of the test (alternative hypothesis $\rho_{\text{RECON}} < \rho_{\text{REAL}}$), and reported the number of genes with significant deterioration at a Bonferroni-corrected threshold of $P < 0.05$.

## S1.3 Association Testing Framework

**Mixed Model for Association Testing.** We employed a linear mixed model framework to assess genetic associations with histological traits. Specifically, for a genotype vector $\boldsymbol{g}$ across $N$ individuals, the $N \times L$ matrix of individual-level embeddings $\boldsymbol{X}$, and the $N \times K$ covariate matrix $\boldsymbol{F}$ of $K$ covariates, we utilized the following generalized linear mixed model:

$$\text{link}^{-1}(\boldsymbol{g}) = \boldsymbol{F}\boldsymbol{\alpha} + \boldsymbol{u}, \quad \text{where}\, \boldsymbol{u} \sim \mathcal{N}(\boldsymbol{0}, \sigma_X^2 \mathcal{K}(\boldsymbol{X})), \tag{S1}$$

where $\boldsymbol{\alpha}$ represents the effects of covariates, and $\mathcal{K}(\boldsymbol{X})$ is an $N \times N$ covariance function that models pairwise similarities between individuals based on their histological embeddings $\boldsymbol{X}$. The link function connects the predicted values to the genotype vector $\boldsymbol{g}$, converting the relationship between them.

**Choice of Likelihood.** We evaluated two likelihood functions for this analysis:

1. *Binomial (two trials):* This method, previously used for modeling genotype minor allele counts [8, 6], leverages a binomial distribution with two trials, suitable for genotypes often represented as counts of minor alleles (0, 1, or 2). The logistic sigmoid function acts as the link function, outputting the rate of success (modeling the variant allele frequency), with the linear mixed model in Eq. (S1) operating on its logits.

2. *Gaussian:* This approach abstracts the discrete nature of genotypes by employing a Gaussian distribution. In this context, the model simplifies to:

$$\boldsymbol{g} \sim \mathcal{N}\left(\boldsymbol{F}\boldsymbol{\alpha}, \sigma_X^2 \mathcal{K}(\boldsymbol{X}) + \sigma_n^2 \boldsymbol{I}_N\right). \tag{S2}$$

As our experiments confirmed sufficient calibration and power of the Gaussian likelihood approach, we opted for it in all experiments.

**Choice of Kernel.** The covariance function $\mathcal{K}(\boldsymbol{X})$ produces an $N \times N$ covariance matrix that characterizes the relationships between individuals based on the histological embeddings. A commonly used function is the linear covariance: $\mathcal{K}_{\text{linear}}(\boldsymbol{X}) = \boldsymbol{X}\boldsymbol{X}^T$ [15, 10], which models linear effects of the embeddings $\boldsymbol{X}$, similar to Bayesian linear regression. However, we observed that using a cosine similarity function provided better calibration of P values [12], ensuring unit diagonal elements of the output covariance. This covariance can be expressed as a linear covariance of transformed features $\tilde{\boldsymbol{X}}$, specifically, $\mathcal{K}_{\text{linear}}(\boldsymbol{X}) = \tilde{\boldsymbol{X}}\tilde{\boldsymbol{X}}^T$, where each row of $\tilde{\boldsymbol{X}}$ is obtained by normalizing the corresponding row of $\boldsymbol{X}$ to have an $L^2$ norm of 1.

**Score Test.** To test for association between a single genetic variant and histological embeddings, we assessed $\sigma^2_{\boldsymbol{X}} > 0$ in the model (Eq. (S1)) using a score test [15, 10]. For the Gaussian model, the test statistic is given by:

$$Q = \frac{1}{2}\boldsymbol{g}^T \boldsymbol{P} \mathcal{K}(\boldsymbol{X}) \boldsymbol{P} \boldsymbol{g}, \tag{S3}$$

where:

$$\boldsymbol{P} = \frac{1}{\hat{\sigma^2_n}} \left( \boldsymbol{I} - \boldsymbol{F} \left( \boldsymbol{F}^T \boldsymbol{F} \right)^{-1} \boldsymbol{F}^T \right), \tag{S4}$$

and $\hat{\sigma^2_n}$ is the maximum likelihood estimator of $\sigma^2_n$ under the null model in Eq. (S2) (with $\sigma^2_{\boldsymbol{X}} = 0$). Asymptotically, the test statistic $Q$ follows a mixture of $\chi^2$ distributions under the null hypothesis:

$$Q \sim \sum_i \phi_i \chi^2_1, \tag{S5}$$

where:

$$\boldsymbol{\phi} = \text{eigenvalues} \left( \frac{1}{2} \boldsymbol{P}^{\frac{T}{2}} \mathcal{K}(\boldsymbol{X}) \boldsymbol{P}^{\frac{1}{2}} \right). \tag{S6}$$

Full details of this derivation can be found in [10]. P values are obtained using the Davies method [4]. Following [15], we use Liu saddlepoint approximation [11] to obtain P values when the Davies method fails to converge.

**Efficient Implementation.** To ensure that HistoGWAS is scalable to large cohorts, we exploit the fact that the dimensionality of the embeddings is typically much lower than the number of individuals ($L \ll N$) [14]. This allows us to achieve linear scaling with respect to the number of individuals. The test statistic $Q$ is computed as follows:

$$\hat{\boldsymbol{\alpha}} = \left( \boldsymbol{F}^T \boldsymbol{F} \right)^{-1} \boldsymbol{F}^T \boldsymbol{g}, \tag{S7}$$

$$\hat{\boldsymbol{y}} = \left( \boldsymbol{g} - \boldsymbol{F} \hat{\boldsymbol{\alpha}} \right), \tag{S8}$$

$$Q = \frac{1}{2\hat{\sigma^2_n}} \left( \tilde{\boldsymbol{X}}^T \hat{\boldsymbol{y}} \right)^T \left( \tilde{\boldsymbol{X}}^T \hat{\boldsymbol{y}} \right). \tag{S9}$$

Using the cyclic property of eigenvalues, the non-zero eigenvalues of $\frac{1}{2}\boldsymbol{P}^{\frac{T}{2}} \tilde{\boldsymbol{X}} \tilde{\boldsymbol{X}}^T \boldsymbol{P}^{\frac{1}{2}}$ can be obtained as the eigenvalues of the matrix $\boldsymbol{\Lambda} = \frac{1}{2} \tilde{\boldsymbol{X}}^T \boldsymbol{P} \tilde{\boldsymbol{X}}$, which is an $L \times L$ matrix. This matrix can be efficiently computed as follows:

$$\widehat{\boldsymbol{X}} = \boldsymbol{P} \tilde{\boldsymbol{X}} = \tilde{\boldsymbol{X}} - \boldsymbol{F} \left( \boldsymbol{F}^T \boldsymbol{F} \right)^{-1} \boldsymbol{F}^T \tilde{\boldsymbol{X}}, \tag{S10}$$

$$\boldsymbol{\Lambda} = \frac{1}{2} \tilde{\boldsymbol{X}}^T \widehat{\boldsymbol{X}}. \tag{S11}$$

All operations required to compute $Q$ and $\boldsymbol{\Lambda}$ scale linearly with $N$. Furthermore, both the computation of the eigenvalues of $\boldsymbol{\Lambda}$ and the calculation of P values depend on the dimensionality of the embeddings, $L$, rather than on $N$. The eigenvalue computation scales cubically with $L$.

## S1.4 Visualization of Genetic Effects on Histology

To illustrate histological alterations linked to significant genetic variants, we describe the main steps involving latent space mixed models, embedding interpolation, and semantic decoding:

1. **Fit Linear Mixed Model:** We fit a linear mixed model where the vector $\boldsymbol{g}$ ($N \times 1$) of genotype values for a specific variant is modeled as the outcome, influenced by covariates $\boldsymbol{F}$ ($N \times K$) with fixed effects $\boldsymbol{\alpha}$ ($K \times 1$), and individual-level embeddings $\boldsymbol{X}$ ($N \times L$) with random effects $\boldsymbol{\beta}$ ($L \times 1$). Covariates include an intercept, sex, age, the leading four genetic principal components, and one-hot encoded variables for the type of death.

2. **Define Genetic Effect Axis:** After fitting the linear mixed model, the mean of the posterior distribution of the random effect $\boldsymbol{\beta}$ is given by:

$$\widehat{\boldsymbol{\beta}} = \widehat{\sigma^2_{\boldsymbol{X}}} \boldsymbol{X}^T \left( \widehat{\sigma^2_{\boldsymbol{X}}} \boldsymbol{X}\boldsymbol{X}^T + \widehat{\sigma^2_n} \boldsymbol{I}_N \right)^{-1} (\boldsymbol{g} - \boldsymbol{F}\widehat{\boldsymbol{\alpha}}), \tag{S12}$$

where $\widehat{\cdot}$ denotes the maximum likelihood estimator. The vector $\widehat{\boldsymbol{\beta}}$ is an $L \times 1$ vector that defines the phenotypic direction most predictive of the genotype. We name this direction the genetic effect axis, leveraging linear projections of embeddings along this axis to quantify and visualize the affected phenotype.

3. **Projection of Individual-Level Embeddings:** Intuitively, projecting embeddings onto the genetic effect axis yields a score that quantifies phenotypic variation in the direction of genetic effect, thereby aiding interpretation of genotype–phenotype relationships. Specifically, the genetic axis score is obtained through the best linear unbiased predictor (BLUP):

$$\boldsymbol{g}_{\text{BLUP}} = \boldsymbol{X}\widehat{\boldsymbol{\beta}} = \underbrace{\widehat{\sigma^2_{\boldsymbol{X}}} \boldsymbol{X}\boldsymbol{X}^T \left( \widehat{\sigma^2_{\boldsymbol{X}}} \boldsymbol{X}\boldsymbol{X}^T + \widehat{\sigma^2_n} \boldsymbol{I}_N \right)^{-1}}_{\boldsymbol{H}} \underbrace{(\boldsymbol{g} - \boldsymbol{F}\widehat{\boldsymbol{\alpha}})}_{\boldsymbol{g}_{\text{R}}}, \tag{S13}$$

where we introduced the matrix $\boldsymbol{H}$ and residuals $\boldsymbol{g}_{\text{R}}$. To avoid overfitting from this in-sample estimator, we use the leave-one-out (LOO) estimator proposed in [13]:

$$\boldsymbol{g}_{\text{LOO},i} = \frac{\boldsymbol{g}_{\text{BLUP},i} - \boldsymbol{H}_{i,i}\boldsymbol{g}_{\text{R},i}}{1 - \boldsymbol{H}_{i,i}}, \tag{S14}$$

where $\boldsymbol{g}_{\text{BLUP}}$ is the in-sample predictor, and $\boldsymbol{H}_{i,i}$ represents the $i$-th diagonal element of the projection matrix. This leave-one-out estimator is used to compute individual-level genotype axis scores for visualization in **Figure 4** .

4. **Projection of Patch-Level Embeddings:** Since the model is linear in the slide-level embeddings and these embeddings are obtained as a linear average of the patch-level embeddings (via average pooling), we can apply the same projection to all patches to obtain patch-level phenotypic scores $\boldsymbol{s}$:

$$\boldsymbol{s} = \boldsymbol{X}_{\text{patch}}\widehat{\boldsymbol{\beta}}, \tag{S15}$$

where $\boldsymbol{X}_{\text{patch}}$ indicates the #patches $\times L$ matrix of patch-level embeddings.

5. **Interpolate Between Embeddings:** First, we compute extreme embeddings for interpolation as the average of patches at both extremes of the distribution of $\boldsymbol{s}$. Specifically, we average all patches in the 1st-5th percentiles of $\boldsymbol{s}$ to obtain $\boldsymbol{z}_m$, and all patches in the 95th-99th percentiles of $\boldsymbol{s}$ to obtain $\boldsymbol{z}_M$. Next, we linearly interpolate between $\boldsymbol{z}_m$ and $\boldsymbol{z}_M$ as:

$$\boldsymbol{z}(\alpha) = (1 - \alpha)\boldsymbol{z}_m + \alpha\boldsymbol{z}_M, \tag{S16}$$

where $\alpha \in [0, 1]$.

6. **Decode Interpolated Embeddings:** Each interpolated embedding $\boldsymbol{z}(\alpha)$ is decoded using the semantic decoder. Given the stochastic nature of our decoder, multiple visual interpretations can be generated by varying the input noise, allowing for several visualizations of the continuum of histological changes (**Supplementary Figures 10**).

All calculations are linear in the cohort size due to leveraging the fact that the embedding dimension is much smaller than the number of individuals. This is achieved by factorizing all operations appropriately and utilizing the Woodbury identity and matrix determinant lemma to solve linear systems, compute inverses, and determine log determinants [2, 3, 14]. Moreover, fitting the linear mixed model uses the $\delta$ reparameterization introduced in [9], where fast grid search on $\delta$ is achieved leveraging that all MLEs can be computed in closed form for any fixed value of $\delta$. All optimized models are made available.

## S1.5 Power Analysis for HistoGWAS

We simulated 64-dimensional individual-level embeddings as a sum of contributions from covariates, a single genetic variant, and Gaussian noise. Each component was simulated as follows:

1. **Covariate Effects:** We generate covariates $\boldsymbol{F}$ (an $N \times K$ matrix) to capture effects from sex (modeled as a 50/50 Bernoulli distribution), age (uniform distribution between 40 and 80), and the four genetic principal components (random normal distribution). The corresponding effects matrix $\boldsymbol{\alpha}$ $(K \times L)$ is sampled from a random normal distribution. The contribution of covariates is computed as:
$$\boldsymbol{X}_c = \boldsymbol{F} \cdot \boldsymbol{\alpha}, \tag{S17}$$
and normalized to have an average variance of $v_c$ across dimensions (we set $v_c = 20\%$):
$$\boldsymbol{X}_c = \sqrt{\frac{v_c}{\text{mean}(\text{var}(\boldsymbol{X}_c, 0))}}\boldsymbol{X}_c. \tag{S18}$$
2. **Genetic Effects:** We generate a genotype vector $\boldsymbol{g}$ $(N \times 1)$ from a binomial distribution with two trials and minor allele frequencies uniformly distributed between 5% and 20%. The genetic effects matrix $\boldsymbol{\beta}$ $(1 \times L)$ is sampled from an iid random normal distribution. The contribution of the genetic variant is computed as:
$$\boldsymbol{X}_g = \boldsymbol{g} \cdot \boldsymbol{\beta}, \tag{S19}$$
and normalized to have an average variance of $v_g$ across dimensions:
$$\boldsymbol{X}_g = \sqrt{\frac{v_g}{\text{mean}(\text{var}(\boldsymbol{X}_g, 0))}}\boldsymbol{X}_g. \tag{S20}$$
3. **Gaussian Noise:** Gaussian noise $\boldsymbol{X}_n$ $(N \times L)$ is generated as:
$$\boldsymbol{X}_n = \text{np.random.randn}(N, L), \tag{S21}$$
and normalized such that the average variance of $\boldsymbol{X}_c + \boldsymbol{X}_g + \boldsymbol{X}_n$ across dimensions is approximately 1:
$$\boldsymbol{X}_n = \sqrt{\frac{1 - v_c - v_g}{\text{mean}(\text{var}(\boldsymbol{X}_n, 0))}}\boldsymbol{X}_n. \tag{S22}$$

## S2 Supplementary Figures

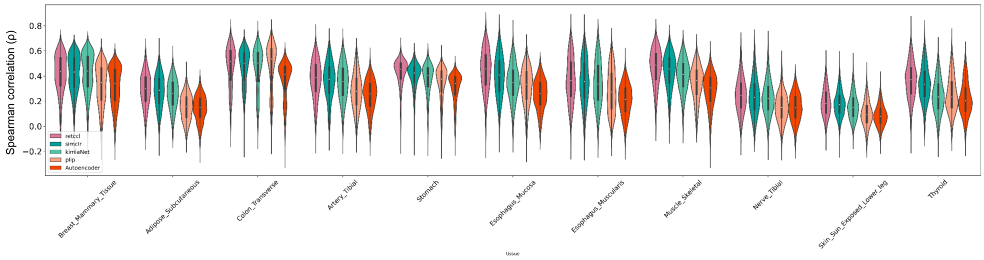


**Fig. S1: Comparative analysis of encoding models and pooling strategies for gene expression prediction.** Shown is the violin plot showing the cross-gene distribution of Spearman correlation ($\rho$) between observed and histologically predicted gene expression levels for test set individuals across the 11 analyzed tissues. This metric quantifies the predictive accuracy of gene expression from individual-level histological embeddings obtained from different models: RetCCL, SimCLR, KimiaNet, PLIP, and Autoencoder (**Methods**). Notably, the RetCCL contrastive learning model exhibits the highest predictive performance across tissues and was selected as HistoGWAS encoder.

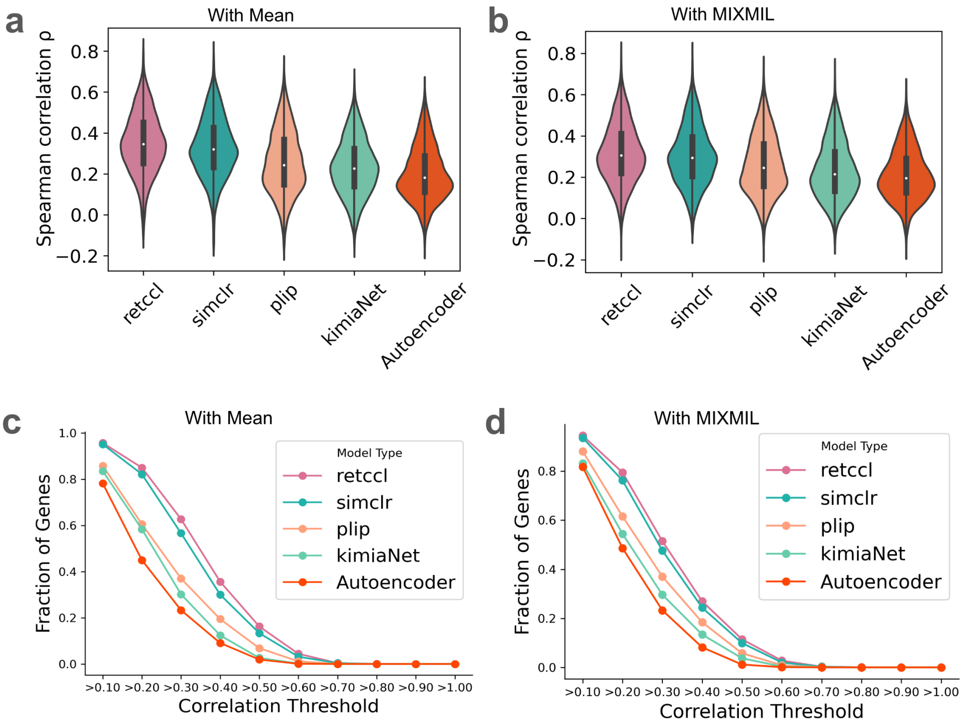


**Fig. S2: Comparison of mean pooling and MixMIL models for predicting gene expression in thyroid tissue.** Panels (**a**) and (**b**) show violin plots of gene-level Spearman correlations ($\rho$) using mean pooling (**a**) or attention-based MixMIL [6] (**b**). Panels (**c**) and (**d**) show the fraction of genes exceeding varying $\rho$ thresholds for mean pooling (**c**) or MixMIL (**d**). Predictive performance and relative ranking are consistent across pooling strategies and metrics, with RetCCL performing best overall.

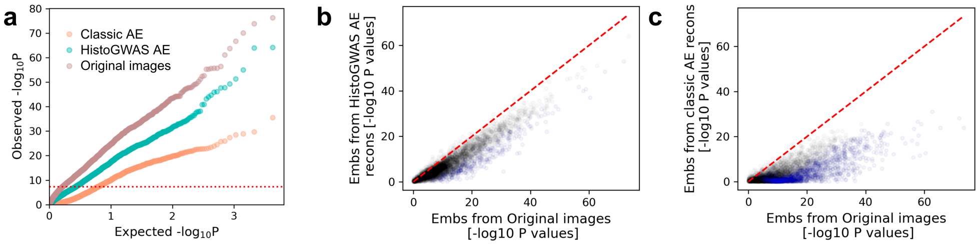


**Fig. S3: Evaluation of semantic autoencoder through expression prediction from reconstructed images in thyroid tissue.** (**a**) QQ plots of $P$ values of association between predicted and observed gene expression levels on test individuals, using embeddings of original images, embeddings of reconstructions from a classic autoencoder (classic AE), or embeddings of reconstructions from HistoGWAS autoencoder (HistoGWAS AE, **Methods**). This evaluation was performed exclusively in thyroid tissue, with the predictive model consistently trained using embeddings of original images on the training set for all comparisons. (**b**) Scatter plot analysis of gene expression prediction statistics ($-\log_{10} P$) employing embeddings of reconstructions from HistoGWAS AE (y-axis) versus embeddings of original images (x-axis) across genes in thyroid tissue. (**c**) Analogous to (**b**), but comparing prediction statistics using embeddings of reconstructions from classic AE (y-axis) versus embeddings of original images (x-axis) across genes in thyroid tissue. Genes highlighted in blue showed a significant deterioration in predictive performance when using embeddings from reconstructed images compared to original images, as determined by Steiger's $Z$ test for dependent correlations (Bonferroni-corrected $P < 0.05$; see **Methods**).

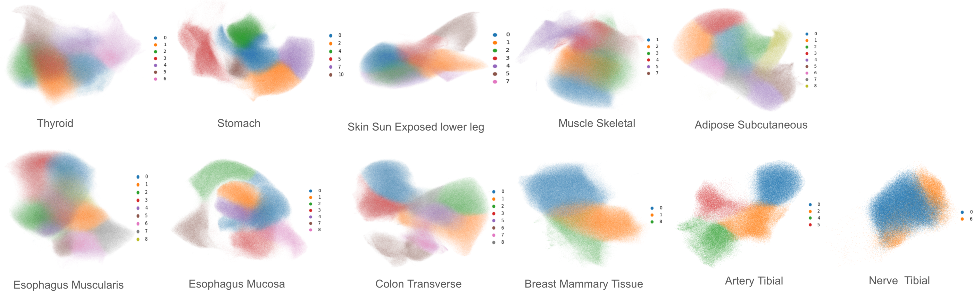


**Fig. S4: Unsupervised data analysis reveals cluster signatures across tissues.** This figure illustrates the results of an unsupervised data analysis, showcasing 68 unique cluster signatures identified across eleven tissues. Shown is the UMAP (Uniform Manifold Approximation and Projection) obtained for each tissue, with colors delineating the distinct cluster signatures as identified through Leiden clustering. Cluster numbering may be non-sequential due to filtering steps retaining only clusters with sufficient representation across slides (**Methods**).

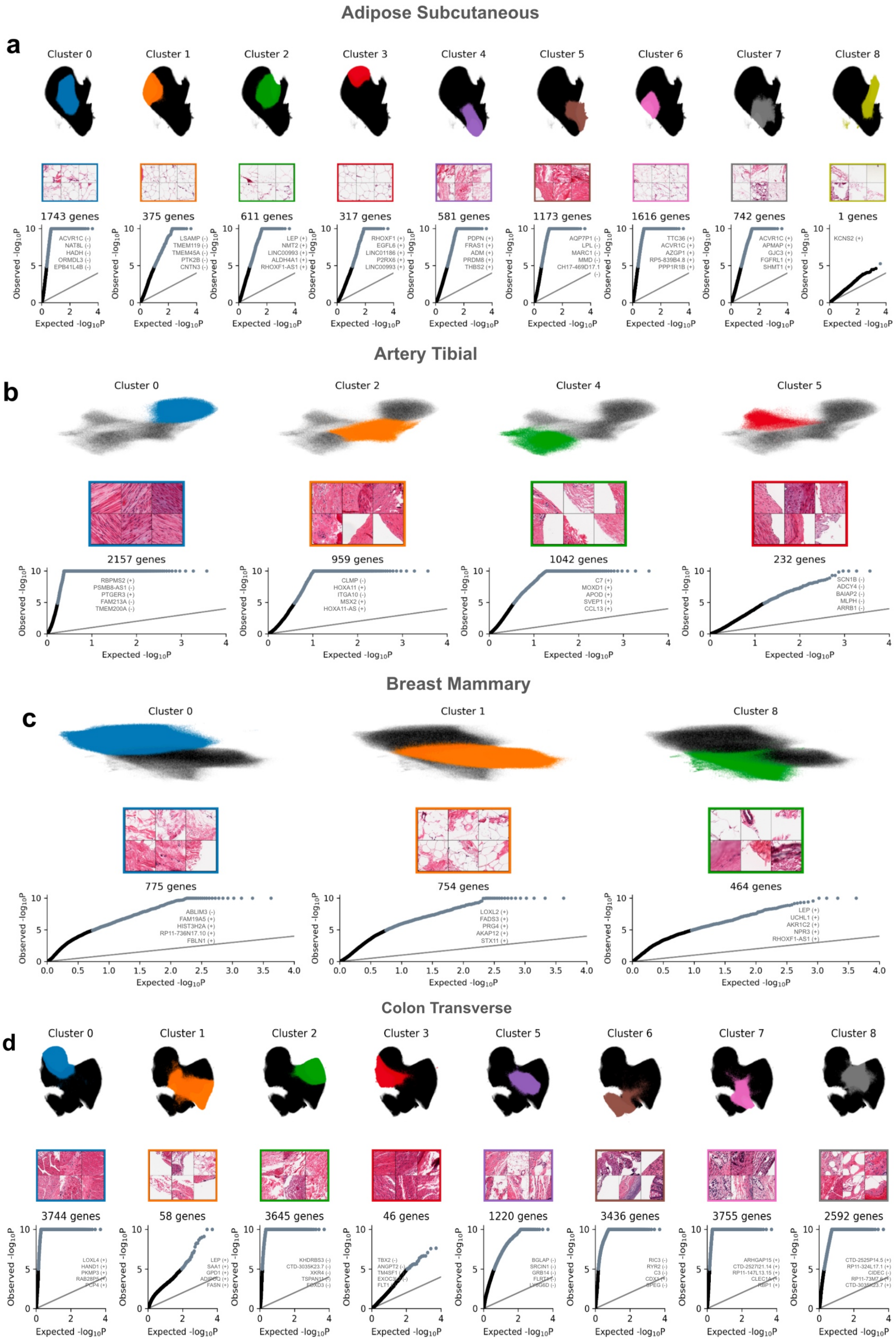


**Fig. S5: Validation of cluster signatures across multiple tissues via gene expression correlation.** *Continued on the next page.*

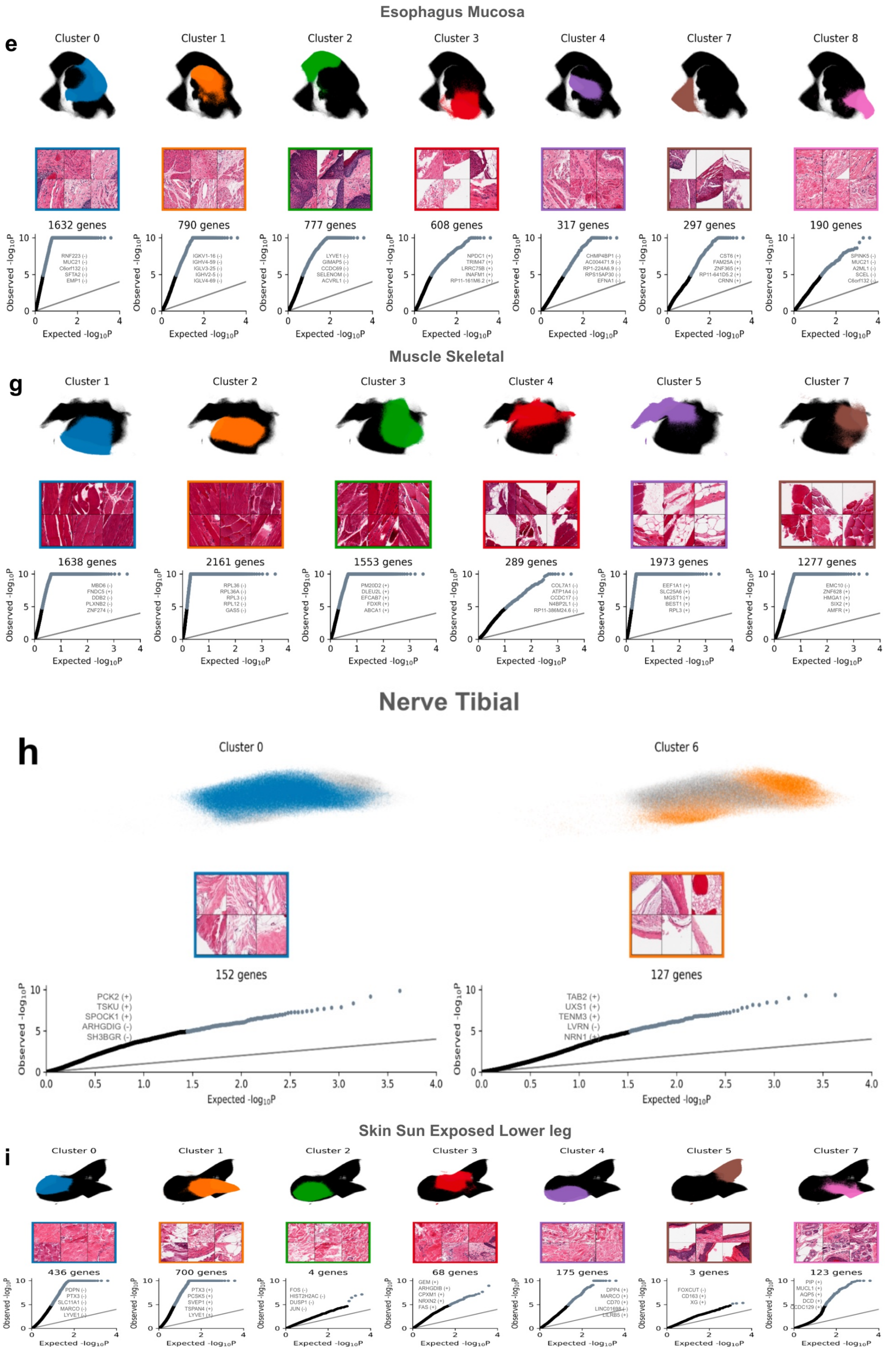


**Fig. S5: Validation of cluster signatures across multiple tissues via gene expression correlation.** *Continued on the next page.*

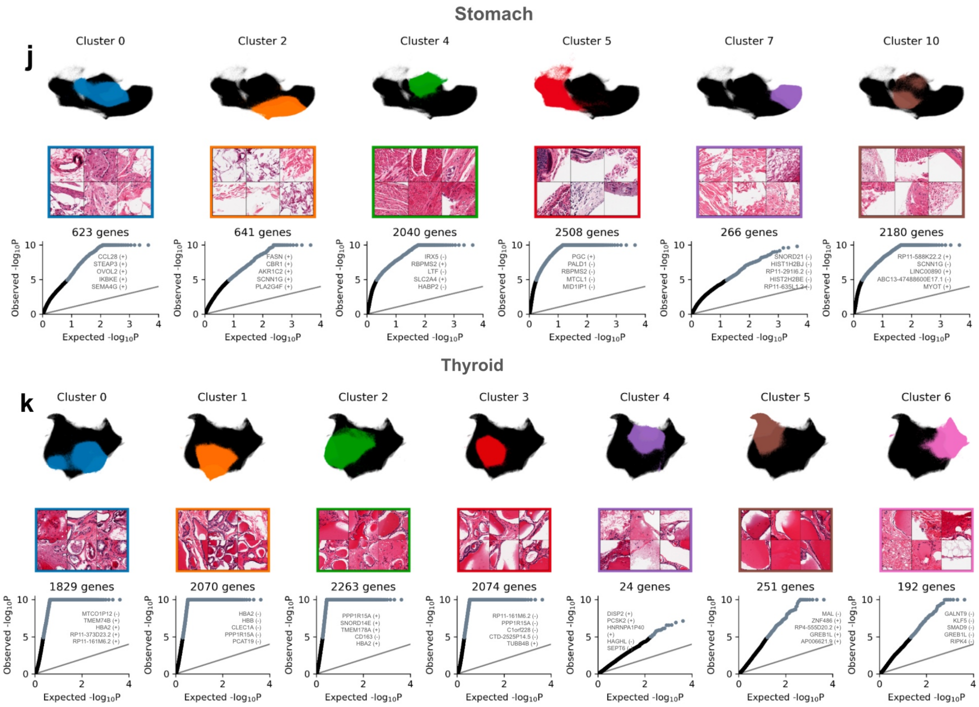


**Fig. S5: Validation of cluster signatures across multiple tissues via gene expression correlation.** This figure illustrates the correlation between the fraction of patches associated with distinct cluster signatures within each slide and their subsequent association with gene expression levels in the corresponding tissue. Shown are 68 cluster signatures across the 11 analyzed tissues: Adipose Subcutaneous (**a**), Artery Tibial (**b**), Breast Mammary Tissue (**c**), Colon Transverse (**d**), Esophagus Mucosa (**e**), Esophagus Muscularis (**f**), Muscle Skeletal (**g**), Nerve Tibial (**h**), Skin Sun Exposed Lower Leg (**i**), Stomach (**j**), and Thyroid (**k**). Uniform Manifold Approximation and Projection (UMAP) visualizations display the distribution of cluster signatures, accompanied by exemplar patches for each tissue type. Quantile-Quantile (QQ) plots of $P$ values underscore the association between cluster signature abundance within slides and gene expression, with the top five genes for each tissue marked, showing the directionality of their expression changes (positive associations marked with (+) and negative with (−)), reflecting over- or underexpression correlated with cluster signature prevalence. Full results from expression correlation analysis can be found in Supplementary Dataset 2. Cluster numbering may be non-sequential due to filtering steps retaining only clusters with sufficient representation across slides (**Methods**).

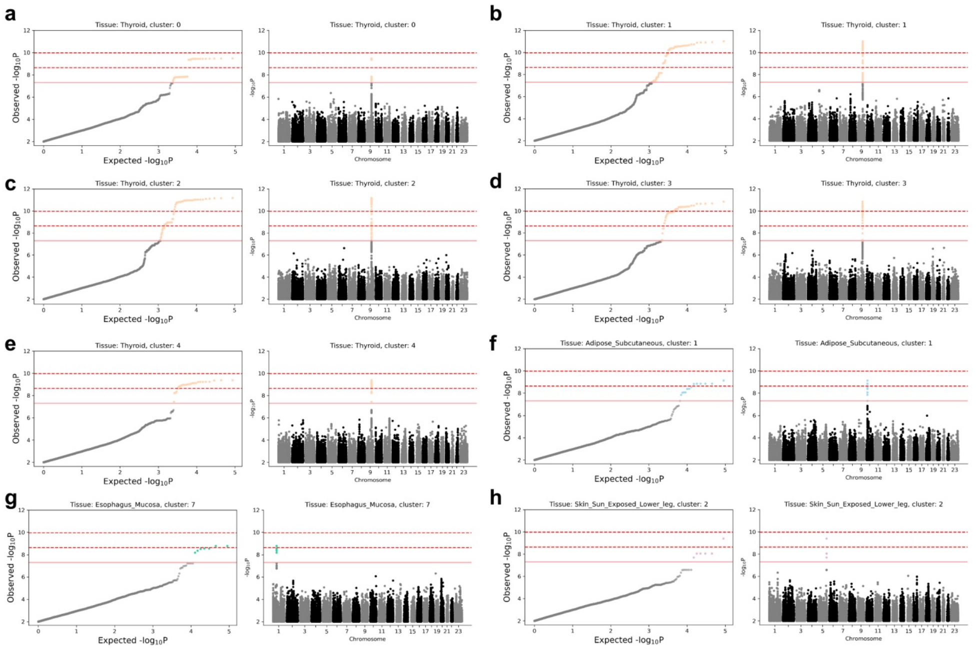


**Fig. S6: Manhattan and QQ plots for cluster signatures with genome-wide significant loci.** Shown are QQ plots (left) and Manhattan plots (right) for cluster signatures with genome-wide significant loci, highlighting five signature clusters in thyroid tissue associated with *rs7030256* (**a**–**e**), one in adipose subcutaneous associated with variant *rs1432621* (**f**), one in esophagus mucosa associated with variant *rs3766325* (**g**), and one in sun-exposed skin associated with *rs1432621* (**h**). We show three $P$ value thresholds for statistical significance: the standard significance level, $P < 5 \times 10^{-8}$, $P < 3.23 \times 10^{-9}$ (corresponding to FWER < 20%, computed through permutations, **Methods**), and $P < 4.29 \times 10^{-10}$ (corresponding to FWER 5%, computed through permutations).

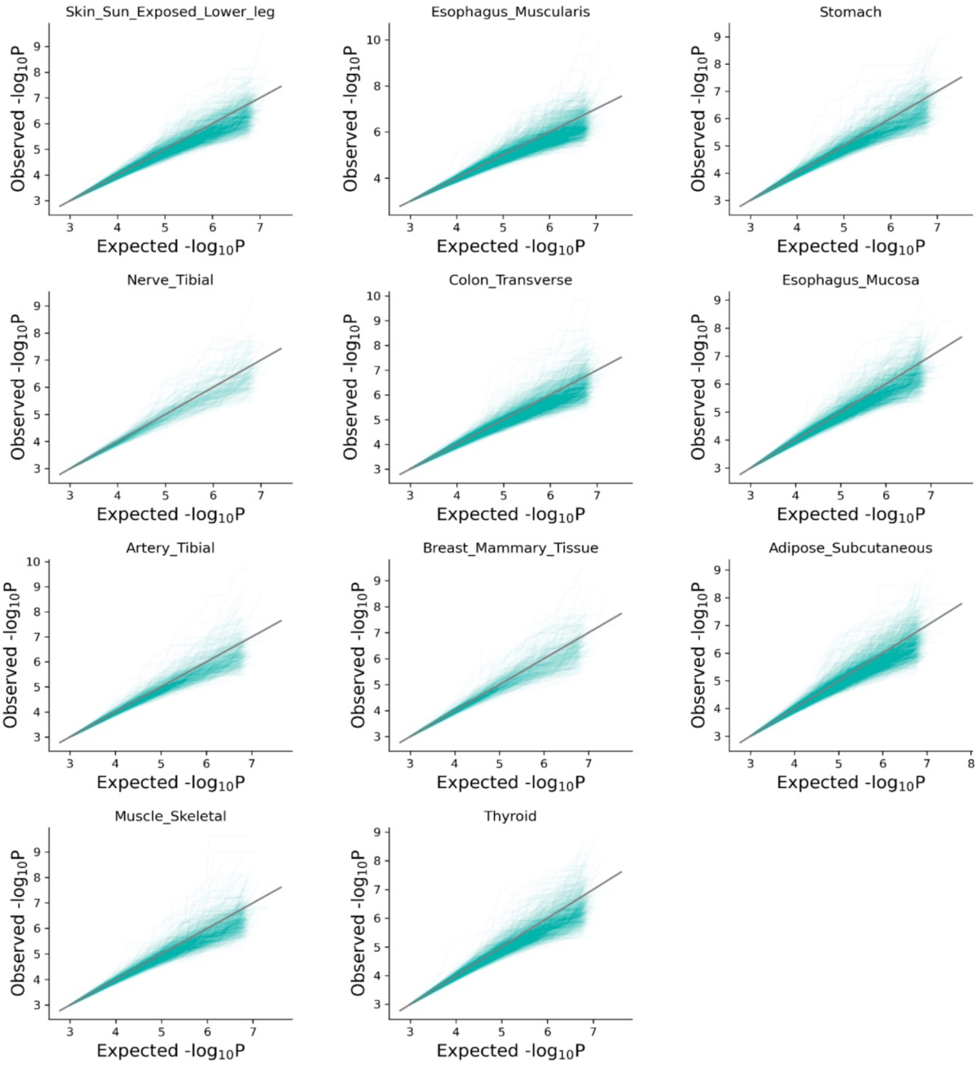


**Fig. S7: Calibration of $P$-values from HistoGWAS under permuted data** Displayed are QQ plots of $P$-values obtained from genome-wide analysis using HistoGWAS for 68 cluster signatures, involving approximately 5 million genetic variants, under permuted data conditions (**Methods**). Each panel illustrates QQ plots across cluster signatures for 100 permutations within a specific tissue.

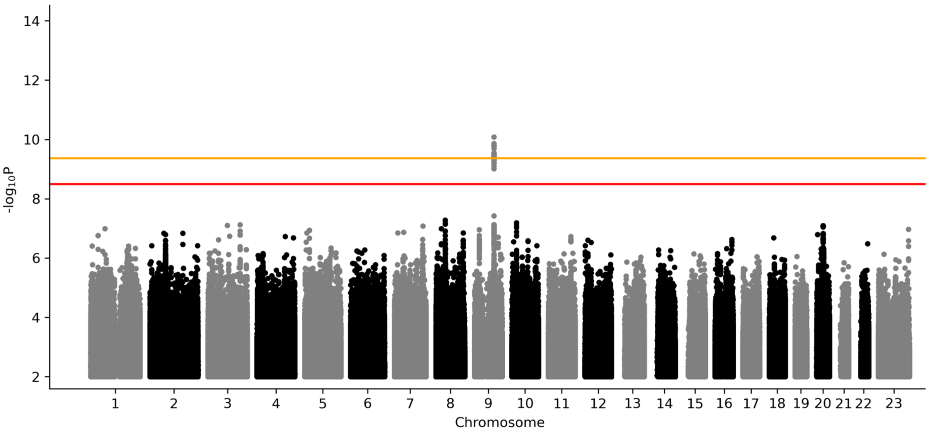


**Fig. S8: GWAS Analysis of Histological Embeddings from Autoencoder Across 68 Cluster Signatures.** Manhattan plot showcasing the P values for genome-wide association studies (GWAS) of 68 cluster signatures. We have the standard significance level ($P < 4.29 \times 10^{-10}$) with red line at family-wise error rates of 5% and the yellow horizontal lines set at family-wise error rates of 20% ($P < 3.23 \times 10^{-9}$), determined through a permutation-based procedure (**Methods**).

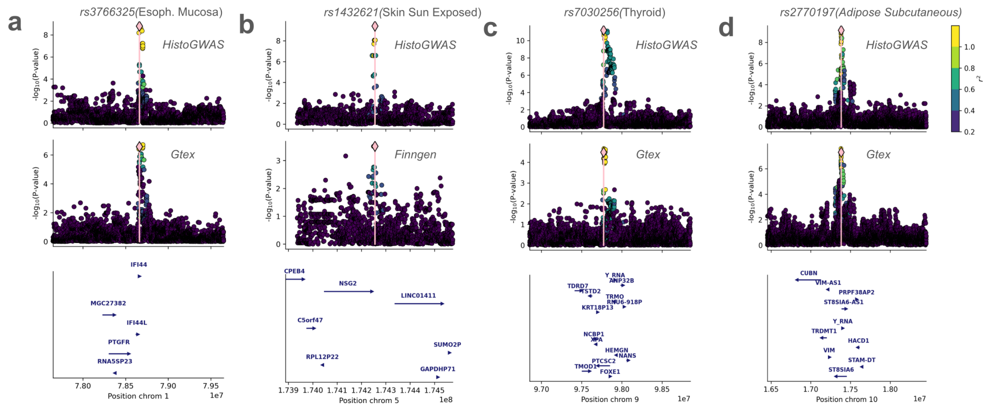


**Fig. S9: Colocalization analyses for four genome-wide significant HistoGWAS loci.** (**a**) shows esophagus mucosa tissueQTL *rs3766325* on chromosome 1 colocalizing with an eQTL signal for *IFI44* (PP-H4 = 0.98). (**b**) shows skin tissueQTL *rs1432621* on chromosome 5 colocalizing with the FinnGen code “Other arthrosis” (PP-H4 = 0.79). (**c**) shows thyroid tissueQTL *rs7030256* on chromosome 9 colocalizing with an eQTL for *FOXE1* (PP-H4 = 0.94). (**d**) shows adipose subcutaneous tissueQTL *rs2770197* on chromosome 10 colocalizing with an sQTL for *ST8SIA6* (PP-H4 = 0.99).

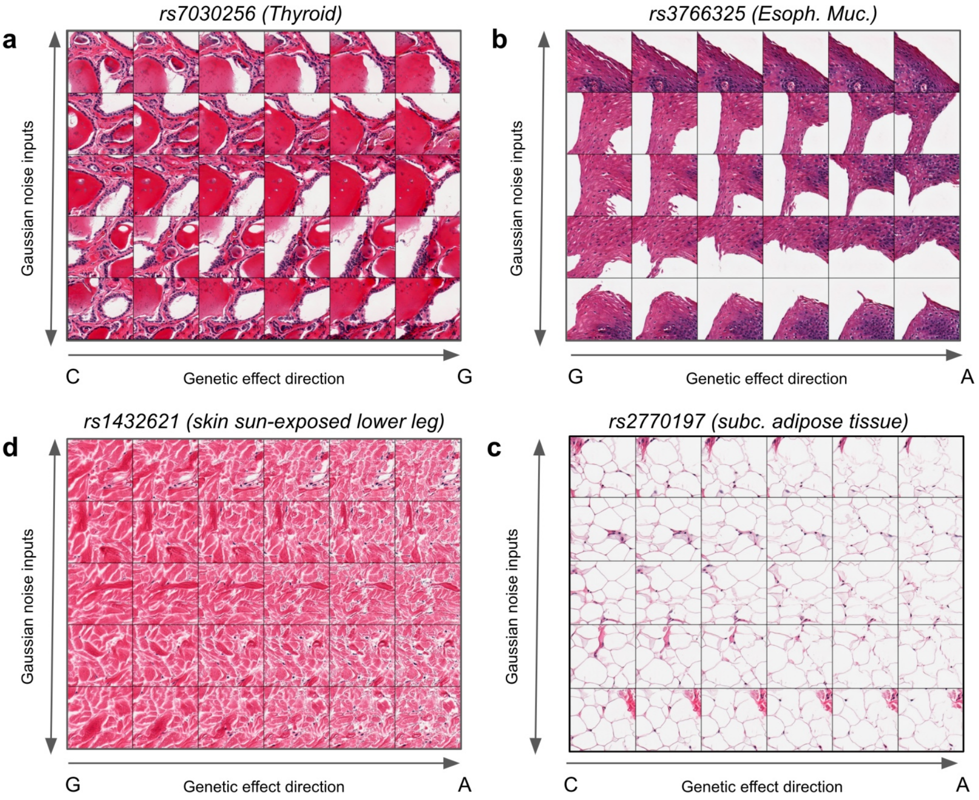


**Fig. S10: Visualization of histological variations induced by genetic variants across different decoder input noises.** This figure demonstrates how varying samples of Gaussian noise input to the semantic decoder influence the visualization of histological changes associated with the four detected genome-wide significant loci. Each panel displays a range of histological outcomes that reflect the stochastic nature of the decoding process, enabling thorough evaluation (**Methods**).

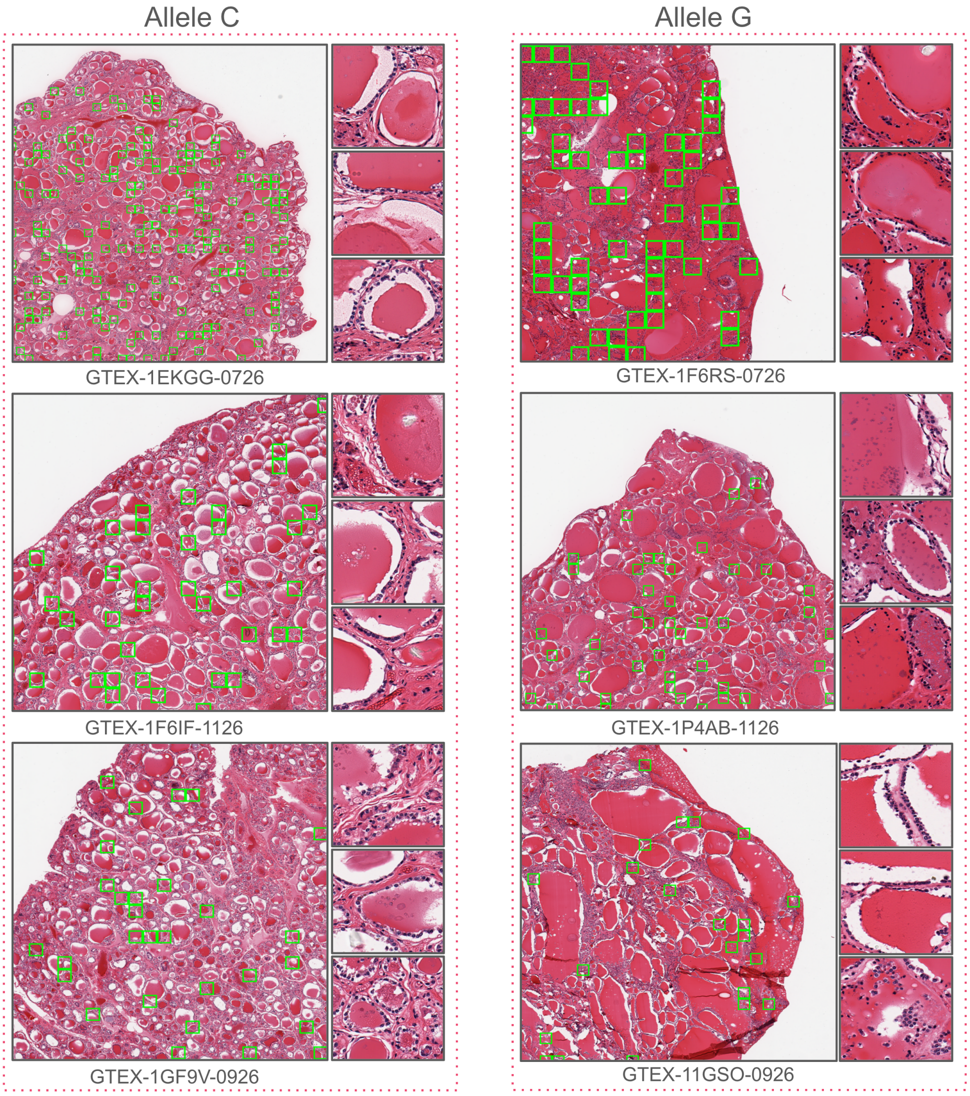


**Fig. S11: Thyroid whole-slide images illustrating extreme phenotypes along the *rs7030256* genetic effect axis.** Extended version of **Figure 4d**. Whole-slide images were selected from samples containing many patches with extreme values along the *rs7030256* genetic effect axis—specifically, at least 40 patches within the top or bottom 5% of patch-level scores (left and right, respectively; **Methods**). Patches in these extreme ranges are highlighted in green, and three representative regions per slide are magnified to illustrate consistent morphological changes along the axis. This visualization illustrates extreme phenotypes along the genetic effect axis in real tissue context. A detailed view of each GTEx slide is available at `https://gtexportal.org/home/histologyPage` using the respective tissue sample IDs.

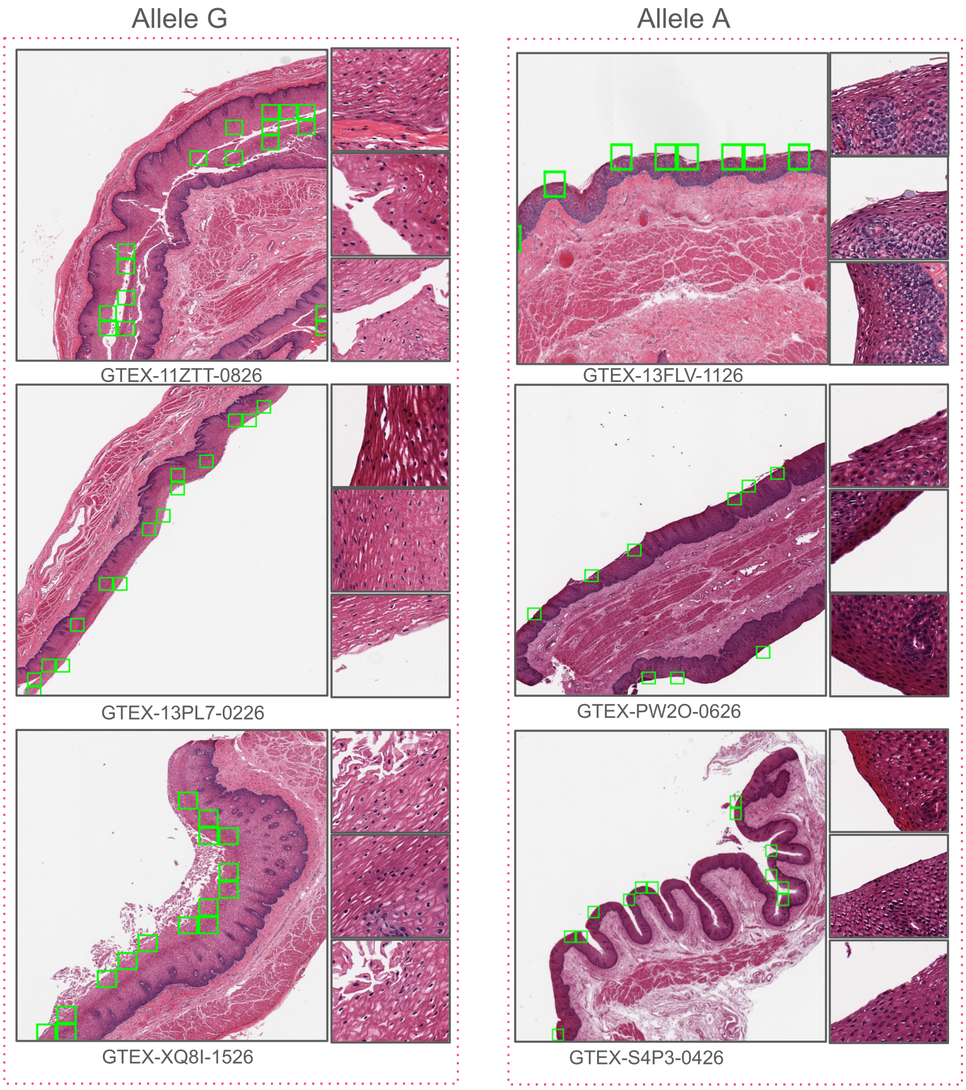


**Fig. S12: Esophagus mucosa image sections illustrating extreme phenotypes along the *rs3766325* genetic effect axis.** Analogous to **Supplementary Figure 11** but for *rs3766325* in esophagus mucosa.

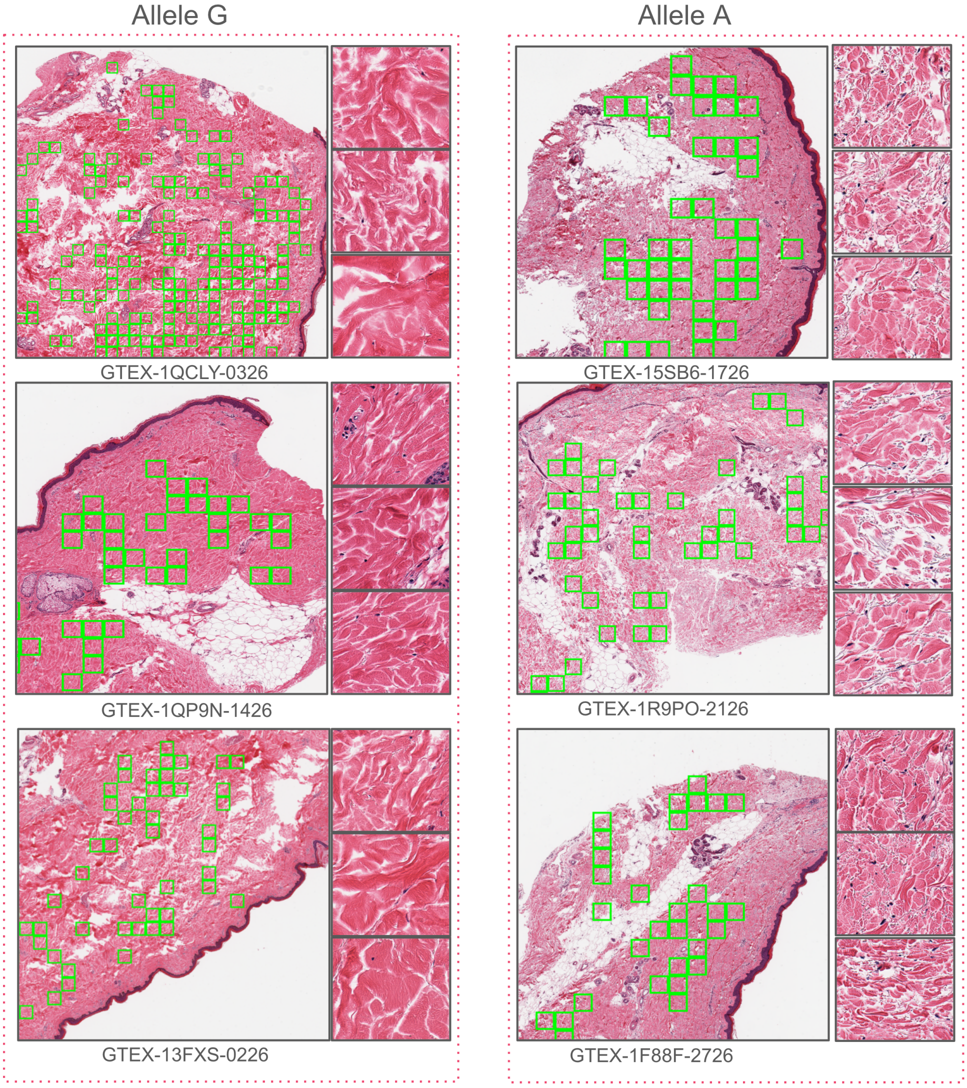


**Fig. S13: Skin image sections illustrating extreme phenotypes along the *rs1432621* genetic effect axis.** Analogous to **Supplementary Figure 11** but for *rs1432621* in skin.

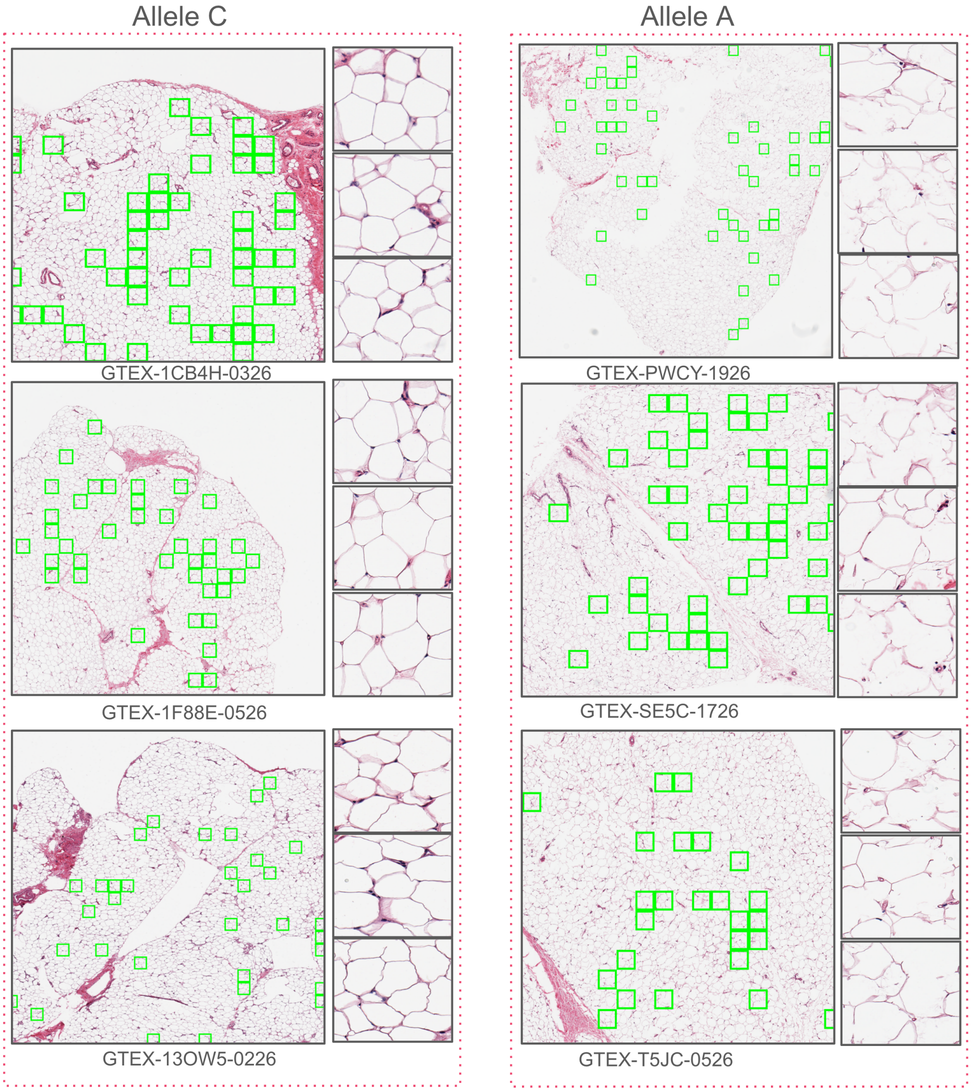


**Fig. S14: Adipose subcutaneous image sections illustrating extreme phenotypes along the *rs2770197* genetic effect axis.** Analogous to **Supplementary Figure 11** but for *rs2770197* in adipose subcutaneous.

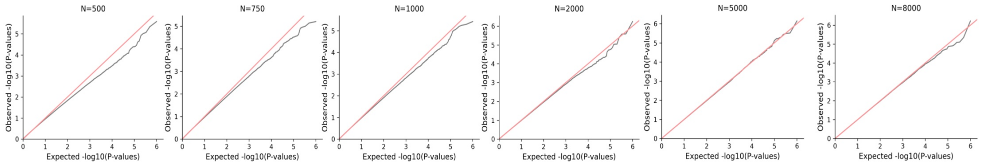


**Fig. S15: Assessment of calibration of HistoGWAS in simulated datasets with no genetic effects.** QQ plots illustrating the calibration of $P$ values from HistoGWAS under null conditions (no genetic effects) across various simulated cohort sizes (**Methods**).

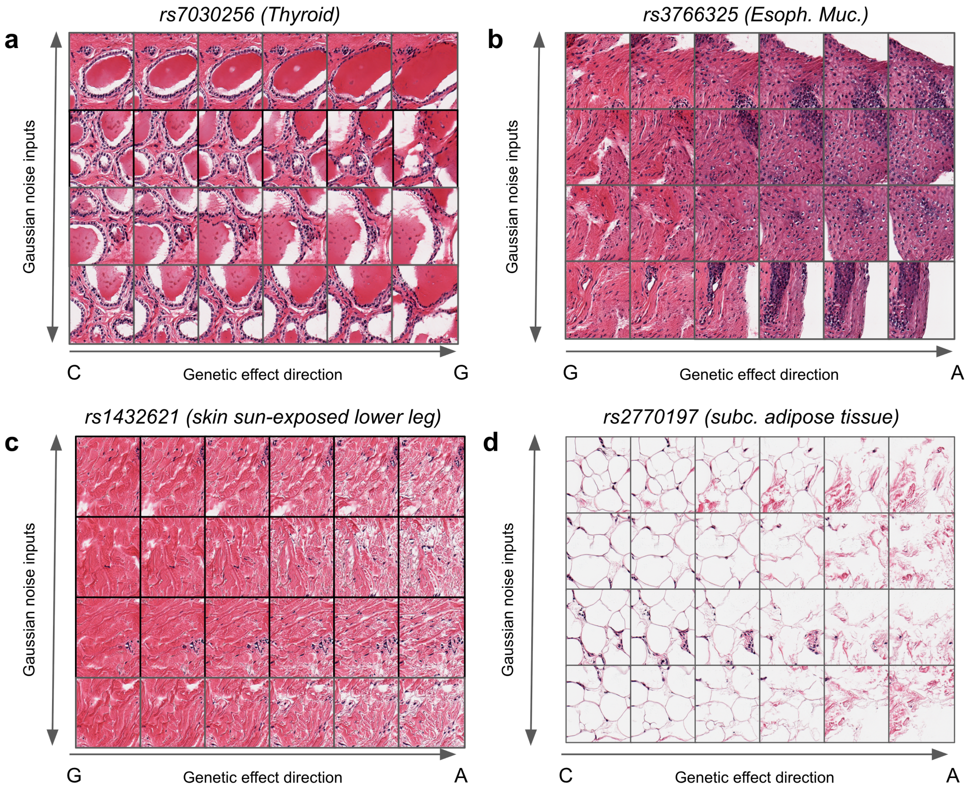


**Fig. S16: Visualization of histological variations induced by genetic variants across tissues using a unified decoder.** This figure illustrates how varying Gaussian noise inputs to a single semantic decoder trained jointly across all tissues influence the visualization of histological changes associated with the four genome-wide significant loci. Each panel presents a spectrum of histological outcomes that highlight both the stochastic nature of the decoding process and the cross-tissue generalization of the unified model (**Methods**).

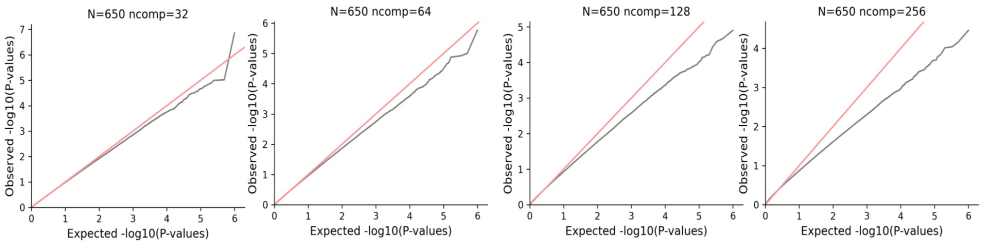


**Fig. S17: Calibration of $P$-values in simulated datasets with no genetic effects across different embedding dimensions.** This figure presents QQ plots of $P$-values derived from HistoGWAS applied to simulated datasets under a null model (no genetic effects), matching the smallest cohort size used in our study ($N = 650$). Each plot varies the number of latent embedding dimensions (ncomps), corresponding to the number of principal components of embeddings (**Methods**). The analysis reveals increasing deflation of $P$-values with a growing number of components, which informed our decision to utilize 64 components in our genetic analyses.

## References


[1] Jordan T Ash, Gregory Darnell, Daniel Munro, and Barbara E Engelhardt. Joint analysis of expression levels and histological images identifies genes associated with tissue morphology. Nature communications, 12(1):1609, 2021.

[2] Francesco Paolo Casale, Barbara Rakitsch, Christoph Lippert, and Oliver Stegle. Efficient set tests for the genetic analysis of correlated traits. Nature methods, 12 (8):755–758, 2015.

[3] Francesco Paolo Casale, Danilo Horta, Barbara Rakitsch, and Oliver Stegle. Joint genetic analysis using variant sets reveals polygenic gene-context interactions. PLoS genetics, 13(4):e1006693, 2017.

[4] Robert B Davies. The distribution of a linear combination of $\chi 2$ random variables. Journal of the Royal Statistical Society Series C: Applied Statistics, 29(3):323–333, 1980.

[5] Birk Diedenhofen and Jochen Musch. cocor: A comprehensive solution for the statistical comparison of correlations. PloS one, 10(4):e0121945, 2015.

[6] Jan P Engelmann, Alessandro Palma, Jakub M Tomczak, Fabian Theis, and Francesco Paolo Casale. Mixed models with multiple instance learning. In International Conference on Artificial Intelligence and Statistics, pages 3664–3672. PMLR, 2024.

[7] Arthur S Goldberger. Best linear unbiased prediction in the generalized linear regression model. Journal of the American Statistical Association, 57(298):369–375, 1962.

[8] Wei Hao, Minsun Song, and John D Storey. Probabilistic models of genetic variation in structured populations applied to global human studies. Bioinformatics, 32(5), 2016.

[9] Christoph Lippert, Jennifer Listgarten, Ying Liu, Carl M Kadie, Robert I Davidson, and David Heckerman. Fast linear mixed models for genome-wide association studies. Nature methods, 8(10):833–835, 2011.

[10] Christoph Lippert, Jing Xiang, Danilo Horta, Christian Widmer, Carl Kadie, David Heckerman, and Jennifer Listgarten. Greater power and computational efficiency for kernel-based association testing of sets of genetic variants. Bioinformatics, 30(22):3206–3214, 2014.

[11] Huan Liu, Yongqiang Tang, and Hao Helen Zhang. A new chi-square approximation to the distribution of non-negative definite quadratic forms in non-central normal variables. Computational Statistics & Data Analysis, 53(4):853–856, 2009.

[12] Christopher D. Manning, Prabhakar Raghavan, and Hinrich Schütze. Introduction to Information Retrieval. Cambridge University Press, Cambridge, England, 2012. doi: 10.1017/CBO9780511809071.

[13] Joel Mefford, Danny Park, Zhili Zheng, Arthur Ko, Mika Ala-Korpela, Markku Laakso, Päivi Pajukanta, Jian Yang, John Witte, and Noah Zaitlen. Efficient estimation and applications of cross-validated genetic predictions to polygenic

risk scores and linear mixed models. Journal of Computational Biology, 27(4): 599–612, 2020.

[14] Rachel Moore, Francesco Paolo Casale, Marc Jan Bonder, Danilo Horta, Lude Franke, Inês Barroso, and Oliver Stegle. A linear mixed-model approach to study multivariate gene–environment interactions. Nature genetics, 51(1):180–186, 2019.

[15] Michael C Wu, Seunggeun Lee, Tianxi Cai, Yun Li, Michael Boehnke, and Xihong Lin. Rare-variant association testing for sequencing data with the sequence kernel association test. The American Journal of Human Genetics, 89(1):82–93, 2011.
